# Supplementary material for: Estimating mutation rates under heterogeneous stress responses
Source: PLoS Comput Biol. 2024 May 28;20(5):e1012146. doi: 10.1371/journal.pcbi.1012146 (PMC11161091; doi:10.1371/journal.pcbi.1012146)
Supplement: S1 File — Mathematical derivations, example mutant count distributions, sensitivity analysis, 95% confidence intervals, parameter estimation and model selection for additional parameter ranges, and comparison of model selection procedures. (PDF) [file pcbi.1012146.s001.pdf]

Supplementary information: Estimating mutation rates under heterogeneous stress responses

Lucy Lansch-Justen[1,*], Meriem El Karoui[2,3,4], and Helen K. Alexander[1,3,*]

May 17, 2024

[1]Institute of Ecology and Evolution, School of Biological Sciences, University of Edinburgh, Edinburgh, Scotland, United Kingdom
[2]Institute of Cell Biology, School of Biological Sciences, University of Edinburgh, Edinburgh, Scotland, United Kingdom
[3]Centre for Engineering Biology, University of Edinburgh, Edinburgh, Scotland, United Kingdom
[4]Bacterial Systems Biology and Anti Microbial Resistance, Laboratoire de Biologie et Pharmacologie Appliquée, École Normale Supérieure Paris-Saclay, Gif-sur-Yvette, France

*Corresponding authors
Email: lucy.lanju@googlemail.com (LLJ), helen.alexander@ed.ac.uk (HKA)

A Deterministic treatment of response-*on* non-mutants

In Model and Methods, we derive the population sizes of the response-*off* and response-*on* non-mutants as

$$n_{off}(t) = n_{off}(0)\mathrm{e}^{(\gamma^s_{off}-\delta^s_{off}-\alpha)t} \tag{1}$$

$$n_{on}(t) = \frac{\alpha n_{off}(0)}{\gamma^s_{off}-\delta^s_{off}-\alpha-(\gamma_{on}-\delta_{on})}\left(\mathrm{e}^{(\gamma^s_{off}-\delta^s_{off}-\alpha)t}-\mathrm{e}^{(\gamma_{on}-\delta_{on})t}\right)+n_{on}(0)\mathrm{e}^{(\gamma_{on}-\delta_{on})t} \tag{2}$$

which assumes that the response-*on* subpopulation can be treated deterministically. However, for small initial population sizes $n_{on}(0)$, this assumption might not hold. Therefore, we test its validity using stochastic simulations; we simulate switching *on* of the response

as a time-inhomogeneous Poisson process with rate $\alpha n_{\mathit{off}}(t)$, the growth dynamics of the response-*on* subpopulation as a continuous-time linear birth-death process with rates

$$\begin{cases} N_{on} \to N_{on}N_{on}, & \text{rate } \gamma_{on} \\ N_{on} \to \varnothing, & \text{rate } \delta_{on} \end{cases} \tag{3}$$

until it reaches a size of $N = 10^4$, and according to Eq 2 afterwards. Then, we compare the resulting population size with Eq 2 at early and late time points: t_1, the expected time of the first mutation (which follows an exponentially-distributed waiting time) in the response-*off* subpopulation, given by

$$t_1 = \frac{\Gamma\left(0, \frac{\mu_{\mathit{off}} n_{\mathit{off}}(0)}{\gamma^s_{\mathit{off}} - \delta^s_{\mathit{off}} - \alpha}\right)}{\gamma^s_{\mathit{off}} - \delta^s_{\mathit{off}} - \alpha}, \tag{4}$$

and t_N, the time when the response-*off* subpopulation reaches a size of $N = 10^9$, given by

$$t_N = \frac{\log\left(\frac{10^9}{n_{\mathit{off}}(0)}\right)}{\gamma^s_{\mathit{off}} - \delta_{\mathit{off}} - \alpha}. \tag{5}$$

For two initial population sizes of the response-*on* subpopulation, (a) $n_{on}(0) = 0$ and (b) $n_{on}(0) = \frac{\alpha}{\gamma^s_{\mathit{off}} - \delta^s_{\mathit{off}}} \cdot n_{\mathit{off}}(0)$, which is a lower bound for the equilibrium fraction of the response-*on* subpopulation, we calculate the error relative to the median of $R = 100$ simulations and the coefficient of variation across the simulations (Fig A). We find that both relative error and coefficient of variation are larger at the earlier than the later time point and that they increase with decreasing relative switching rate $\tilde{\alpha} = \frac{\alpha}{\gamma^s_{\mathit{off}}}$ and increasing relative fitness of response-*on* cells $r_{on} = \frac{\gamma_{on} - \delta_{on}}{\gamma^s_{\mathit{off}} - \delta^s_{\mathit{off}}}$. In general, Eq 2 tends to slightly overestimate the size of the response-*on* subpopulation (indicated in red), but the error is with $< 1\,\%$ sufficiently small to justify our approximation.

B Example mutant count distributions

In the model selection in the main Results, we select between the homogeneous-response and the heterogeneous-response models and find that, in many cases, both models fit the simulated mutant count data similarly well. Here, to gain insight into why this is the case, we show example mutant count distributions using the same parameters as in respective main Results section. This includes distributions under permissive conditions using the standard model (Fig BI) and under stressful conditions for both homogeneous-response (Fig BII) and heterogeneous-response (Fig BIII) models. We consider parameter settings such that the increase in population mean mutation rate is comparable in both the homogeneous and heterogeneous cases ($\frac{\mu^s}{\mu^p} = \bar{M} = 5$).

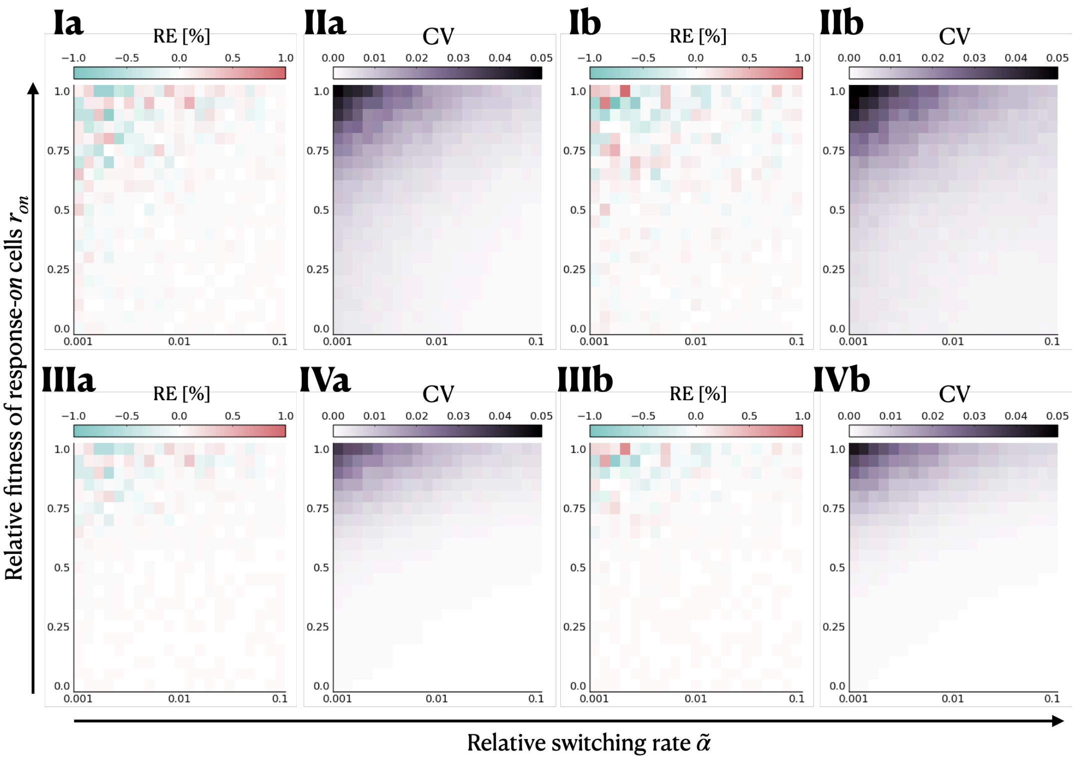

Figure A: **Response-*on* non-mutants can be treated deterministically.** Deterministic compared to stochastic dynamics of response-*on* non-mutants for initial population sizes of the response-*on* subpopulation of (**a**) $n_{on}(0) = 0$ and (**b**) $n_{on}(0) = \frac{\alpha}{\gamma^s_{off} - \delta^s_{off}} \cdot n_{off}(0)$. We simulate switching *on* of the response as a time-inhomogeneous Poisson process and the dynamics of response-*on* non-mutants as birth-death processes ($R = 100$ simulation runs). Then, we compare the resulting population sizes with Eq 2 at early and late time points. **I** Relative error of the median in % at t_1, **II** coefficient of variation across the simulations at t_1, **III** relative error of the median in % at t_N and **IV** coefficient of variation across the simulations at t_N. The parameters used in the simulations are $\gamma^s_{off} = 1\,h^{-1}$ $\delta^s_{off} = \delta_{on} = 0\,h^{-1}$, $\alpha \in [0.001,\ 0.1]\ h^{-1}$, $\gamma_{on} \in [0.0,\ 1.0]\ h^{-1}$.

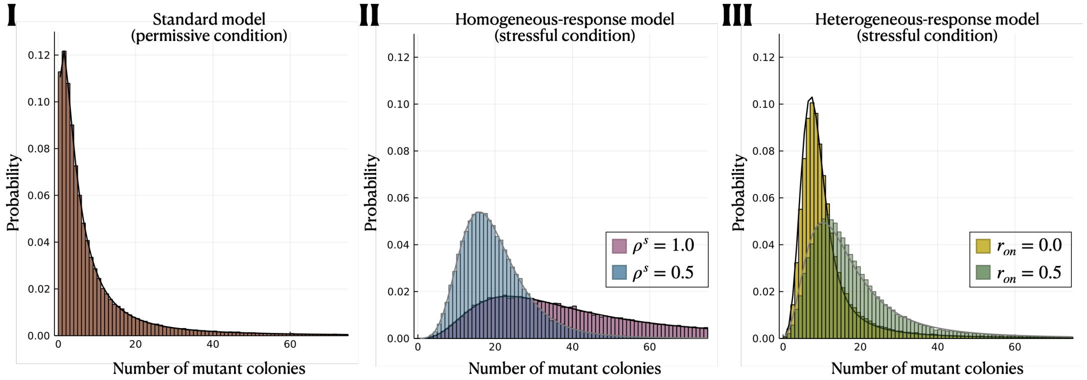

Figure B: **Example mutant count distributions.** We simulate $c = 10^5$ parallel cultures under **I** permissive conditions using the standard model, and under stressful conditions using **II** the homogeneous-response model with an increase in mutation rate of $\frac{\mu^s}{\mu^p} = 5$ and with either no mutant fitness cost ($\rho^s = 1$, purple) or halved fitness ($\rho^s = 0.5$, blue), or **III** the exact heterogeneous-response model with a zero ($r_{on} = 0$, yellow) or intermediate ($r_{on} = 0.5$, dark green) relative fitness of response-*on* cells. For the heterogeneous-response model, we simulate with a mutation-rate increase such that the increase in population mean mutation rate is $\bar{M} = 5$. The lines give the analytically derived distributions (approximate in the case of the heterogeneous-response model).

When there is no mutant fitness cost in the homogeneous-response model ($\rho^s = 1$; Fig BII, purple) and response-*on* cells are non-dividing in the heterogeneous-response model ($r_{on} = 0$; Fig BIII, yellow), the mutant count distributions are clearly different. However, when increasing the severity of the mutant fitness cost ($\rho^s = 0.5$; Fig BII, blue) or the division rate of response-*on* cells ($r_{on} = 0.5$; Fig BIII, dark green), the mutant count distributions have a similar shape. At the same time, the analytical mutant count distribution under the approximate heterogeneous-response model (lines in Fig BIII) deviates from the simulated exact distribution (bars), with a greater deviation for larger division rates of response-*on* cells.

C Calculation of the duration of the growth phase

In all simulations to test our inference method, we set the duration t_f of the growth phase such that the expected number of *mutations*, m (not *mutants*) equals one, by numerically

solving the following equations:

$$\text{Permissive conditions: } \mathbb{E}[m] = \int_0^{t_f} \nu_{\mathit{off}}^{p} n_{\mathit{off}}^{p}(t)\,\mathrm{d}t \stackrel{!}{=} 1 \tag{6}$$

$$\text{Stressful conditions: } \mathbb{E}[m] = \int_0^{t_f} \nu_{\mathit{off}}^{s} n_{\mathit{off}}^{s}(t) + \nu_{on} n_{on}(t)\,\mathrm{d}t \stackrel{!}{=} 1 \tag{7}$$

This way, the resulting number of resistant mutant colonies on each selective plate is similar across the considered parameter ranges and usually within an experimentally countable range of zero to a couple hundred. For example, the duration of simulated fluctuation assays under permissive conditions with the default parameter settings (main text Table 2) is set to $t_f \approx 9.21\,h$.

D Sensitivity of the estimation to the number of parallel cultures

In the first main Results section, we show that the estimation of the mutation-rate increase $\frac{\mu_{on}}{\mu_{\mathit{off}}}$ in the heterogeneous-response model is accurate and precise for sufficiently large mutation-supply ratio, $\mathcal{S} \sim \mathcal{O}(1)$. Here, we analyse how this result depends on the number of parallel cultures used to estimate $\frac{\mu_{on}}{\mu_{\mathit{off}}}$. We use the same simulation parameters as in the respective main Results section (with relative switching rate $\tilde{\alpha} = 0.05$) but smaller numbers of parallel cultures ($c = 25$, 12, 6, 3). We find that, as expected, our estimation method performs worse for smaller c (Fig CI). Nonetheless, the median relative error approaches zero for increasing simulated mutation-rate increase (Fig CII), implying that our method remains accurate (i.e. unbiased) independent of the number of parallel cultures used. However, the coefficient of variation across the estimates is consistently larger for smaller c (Fig CIII) and, therefore, precision is lost.

E Width of 95% confidence intervals on parameter estimates

In the first main Results section, we consider maximum likelihood point estimates of the mutation-rate increase $\frac{\mu_{on}}{\mu_{\mathit{off}}}$ from $R = 100$ simulated data sets under the heterogeneous-response model. There, we use the median relative error and the coefficient of variation across these 100 point estimates to measure the accuracy and precision, respectively, of our new inference method. Here, we evaluate our method's performance using 95% confidence intervals on each estimate, from the same simulated data as in the respective main Results section. To summarise the confidence intervals across $R = 100$ estimates, we calculate the median of their normalised width, i.e. the difference between the upper and lower bounds of the confidence interval divided by the maximum likelihood estimate (example in Fig DI). Generally, presenting boxplots of $R = 100$ maximum likelihood point estimates

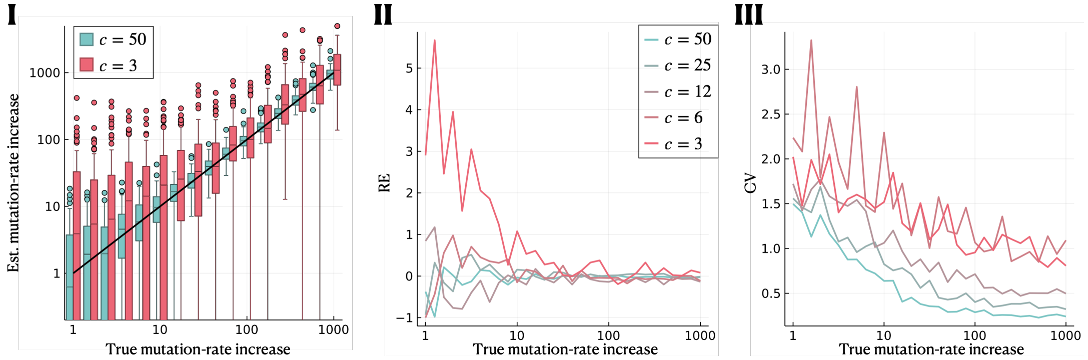

Figure C: **Sensitivity of the estimation to the number of parallel cultures.** Here, we use the same simulation parameters as in Results Fig 2 (where $c = 50$), but consider smaller numbers of parallel cultures ($c = 25, 12, 6, 3$). **I** Estimated compared to true mutation-rate increase using $c = 50$ (red) or $c = 3$ (light blue) parallel cultures. The black line gives the true value of $\frac{\mu_{on}}{\mu_{off}}$. **II** Median relative error and **III** coefficient of variation across the estimates for different numbers of parallel cultures.

(as done throughout the main Results) gives the same qualitative picture as presenting the median normalised width of $R = 100$ confidence intervals (compare Fig DI to Results Fig 2A). At the same time, the median normalised width of the confidence intervals shows a qualitatively similar relationship with $\frac{\mu_{on}}{\mu_{off}}$ and $\tilde{\alpha}$ (Fig DIII) as the coefficient of variation across $R = 100$ point estimates (Results Fig 2C): for a sufficiently large mutation-supply ratio $\mathcal{S} \approx \frac{\mu_{on}}{\mu_{off}}\tilde{\alpha} \sim \mathcal{O}(1)$, confidence intervals are narrow, with a normalised width < 2 (i.e. extending less than 2-fold around the maximum likelihood estimate). Moreover, we confirm that the number of cases in which the true value of $\frac{\mu_{on}}{\mu_{off}}$ lies outside the calculated 95% confidence interval is $< 5\%$ overall, with overestimation being slightly more frequent than underestimation (Fig DII).

F The impact of cell death on parameter estimation for a smaller switching rate

In the second main Results section, we show that the impact of cell death on the estimation of the mutation-rate increase depends on which subpopulation is affected by death. However, estimates remain largely unbiased when all cells are affected equally, although the variation of these estimates increases. This result is not an artefact of the specific parameters used in the respective main Results section: it also holds for different values of the relative switching rate, here shown with $\tilde{\alpha} = 0.01$ (Fig E) instead of $\tilde{\alpha} = 0.05$ (Results Fig 3).

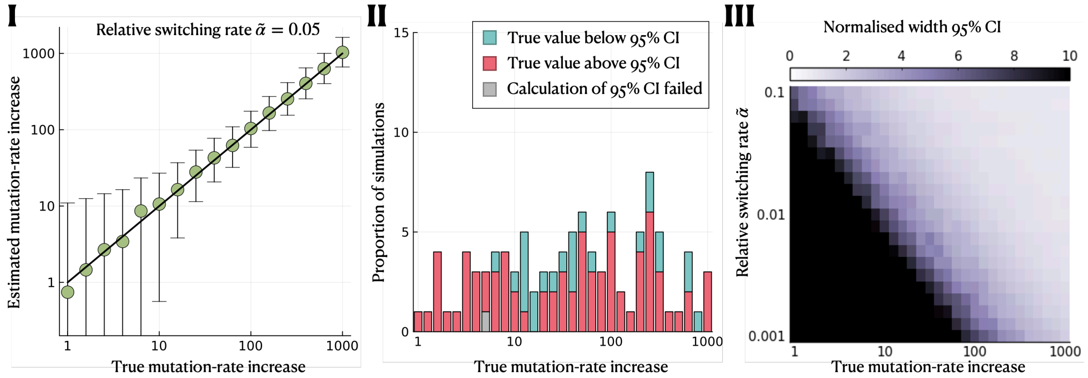

Figure D: 95% **confidence intervals on parameter estimates.** Here, we use the same simulated data as in Results Fig 2 but, additionally, calculate 95% profile likelihood confidence intervals around the maximum likelihood estimates of the mutation-rate increase $\frac{\mu_{on}}{\mu_{off}}$. **I** Median estimated $\frac{\mu_{on}}{\mu_{off}}$ with whiskers extending to the median lower and upper bound of the 95% confidence intervals. **II** Proportion of simulations for which the true value lies outside the 95% confidence interval. **III** Normalised width of 95% confidence intervals.

This result can be explained as follows. Generally, the estimation of μ_{off} is not noticeably impacted by cell death because it is jointly inferred under stressful and permissive conditions, and for the latter, there is no cell death. Therefore, any biases in the estimation of $\frac{\mu_{on}}{\mu_{off}}$ stem from biases in estimating μ_{on}. Now, death in response-*off* cells causes an underestimation of the number of cell divisions to reach the final population sizes, as pointed out in [1]. This, in turn, results in an overestimation of the mutation rate of response-*on* cells because this is calculated as mutations per response-*on* cell, per division in response-*off* cells ($\mu_{on} := \frac{\nu_{on}}{\gamma^s_{on}}$). At the same time, death in response-*on* cells can lead to the extinction of response-*on* mutant lineages, causing an underestimation of $\mathcal{S}$ and, with it, underestimation of μ_{on}. These two effects counteract each other, leading to an unbiased estimate overall.

G The impact of differential mutant fitness on parameter estimation

We next test the robustness of our method to differential mutant fitness, which is neglected in the inference under the heterogeneous-response model. For this purpose, we simulate fluctuation assays under an extended model of heterogeneous stress responses (Results Fig 1C) with differential mutant fitness using a parameter range of $\rho_{off} := \rho^p_{off} = \rho^s_{off} \in [0.0,\ 1.5]$. Note that we consider a differential mutant fitness only in response-*off* cells as

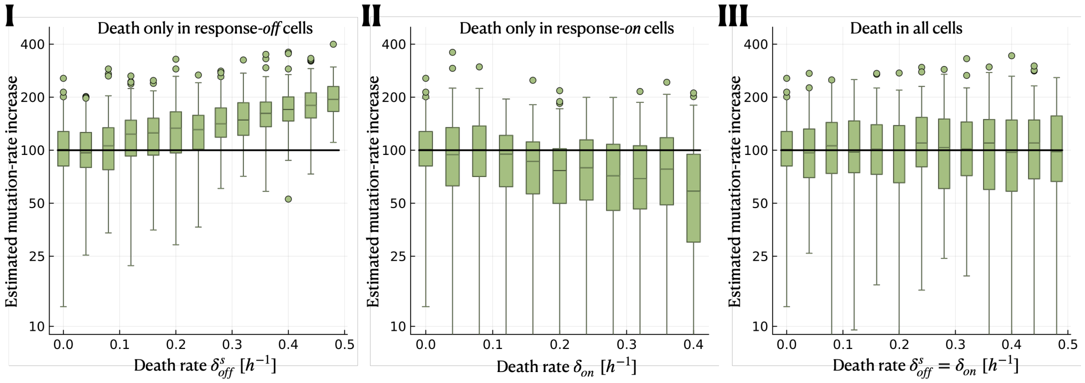

Figure E: **Cell death has limited impact on the estimation of the mutation-rate increase.** Here, we repeat the analysis in Results Fig 3 but with a lower switching rate, α. We simulate using the heterogeneous-response model extended by cell death but neglect cell death in the model used to estimate $\frac{\mu_{on}}{\mu_{off}}$. The black solid lines indicate the true mutation-rate increase used in the simulations. **I** Estimated mutation-rate increase when only response-*off* cells are affected by cell death, **II** when only response-*on* cells are affected by cell death and **III** when all cells are affected by cell death equally. The parameter range used in the simulations is $\alpha = 0.01\ h^{-1}$, $\delta^s_{off} \in [0.0,\ 0.5]\ h^{-1}$, $\delta_{on} \in [0.0,\ 0.5]\ h^{-1}$.

the response-*on* cells have a zero division rate in this set of simulations and are, therefore, unaffected by a differential mutant fitness.

From the resulting mutant count data, we estimate the mutation-rate increase $\frac{\mu_{on}}{\mu_{off}}$ and compare it with the true value to determine any biases caused by neglecting the differential mutant fitness in the inference (Fig F). We find that neglecting this effect in the inference leads to a slight underestimation of the mutation-rate increase when mutations bring a fitness advantage ($\rho_{off} > 1$). Fitness advantages could arise, for example, because the same antibiotic is used as a stressor and on the selective plates or because two different antibiotics are used, but mutants are cross-resistant [2]. However, the bias remains small (relative error of the median $< 17\,\%$) across the tested parameter range. If mutants have a fitness cost ($\rho_{off} < 1$), the mutation-rate increase is estimated accurately.

H Estimation when response-*on* cells have a known non-zero division rate

In the third main Results section, we evaluate the performance of our method in estimating the mutation-rate increase when response-*on* cells have a non-zero division rate for the

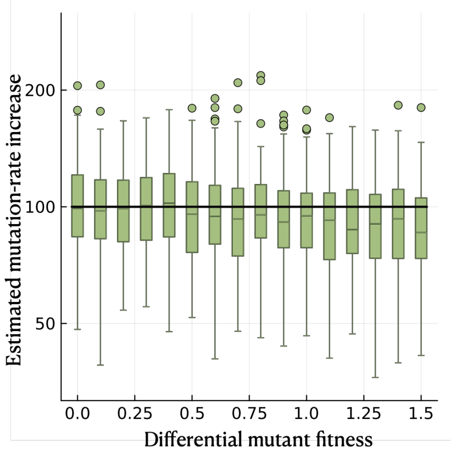

Figure F: **Differential mutant fitness has minimal impact on the estimation of the mutation-rate increase under the heterogeneous-response model.** We simulate using the heterogeneous-response model with mutants having a different division rate than non-mutants but neglect this effect in the model used to estimate $\frac{\mu_{on}}{\mu_{off}}$. The black solid line indicates the true mutation-rate increase. The parameter range used in the simulations is $\rho^p_{off} = \rho^s_{off} \in [0.0,\ 1.5]$.

cases that (i) the non-zero division rate is neglected in the inference (setting $r_{on} = 0$) and (ii) $r_{on} > 0$ is inferred additionally. Here, we consider a third case: r_{on} is set to the true value, which could, for example, be measured in microfluidics experiments using time-lapse microscopy and image analysis to estimate the division rate of cells identified as response-*on*. Interestingly, we find that setting r_{on} to the true value hardly improves the estimation of the mutation-rate increase (Fig G). The reason for this lies in the approximation of the size of the non-mutant response-*on* subpopulation (main text, Eq 13) which assumes that $\gamma_{on} - \delta_{on} \ll \gamma^s_{off} - \delta^s_{off} - \alpha$ and is no longer valid for large $r_{on} = \frac{\gamma_{on}-\delta_{on}}{\gamma^s_{off}-\delta^s_{off}} \to 1$. Therefore, setting r_{on} to the true value still results in a biased estimate. This suggests that, given the available inference method, obtaining a precise estimate of r_{on} from a separate experiment is not worthwhile besides validating that it is small.

I Estimates of the mutant fitness cost in the homogeneous-response model

In the model selection in the main Results, we select between homogeneous and heterogeneous-response models using a two-step selection procedure. Here, we show which version of the homogeneous-response model is chosen by the likelihood-ratio test in the first selection step and what mutant fitness costs are estimated by the chosen model. We find that, when

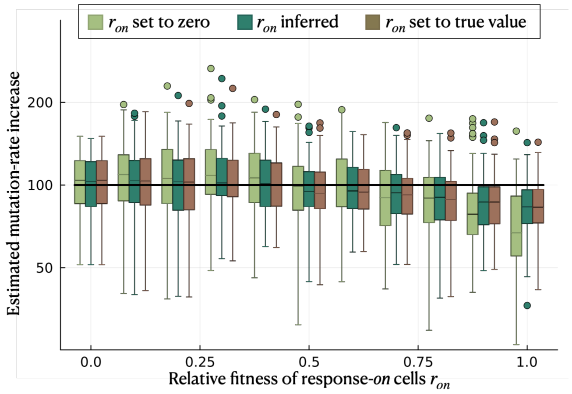

Figure G: **Setting the relative fitness of response-*on* cells to the true value only marginally improves the estimation of the mutation-rate increase.** We simulate using the heterogeneous-response model with $r_{on} \geq 0$ being the relative fitness of response-*on* cells compared to response-*off* cells. We consider three cases for the inference: (i) setting r_{on} to zero and only inferring μ_{off} and $\mathcal{S}$, (ii) inferring r_{on} additionally, and (iii) setting r_{on} to its true value and only inferring μ_{off} and $\mathcal{S}$. From the estimates of μ_{off} and $\mathcal{S}$ we calculate $\frac{\mu_{on}}{\mu_{off}}$. The solid black line indicates the true value of $\frac{\mu_{on}}{\mu_{off}}$. The parameter range used in the simulations is $\gamma_{on} \in [0.0,\ 1.0]\ h^{-1}$.

simulating with small relative fitness of response-*on* cells r_{on}, the homogeneous-response model with unconstrained mutant fitness (ρ^p and ρ^s inferred) is selected in most cases, whereas for large r_{on} the homogeneous-response model without differential mutant fitness ($\rho^p = \rho^s = 1$) is selected in most cases (Fig HI). The homogeneous-response model with constrained mutant fitness ($\rho^p = \rho^s$ inferred) is selected for only a small number of simulations.

For the homogeneous-response model with ρ^p and ρ^s inferred, the mutant fitness under permissive conditions (ρ^p) is correctly estimated as $\approx 0.98 \pm 0.17$. For the mutant fitness under stressful conditions (ρ^s), on the other hand, an increasingly severe mutant fitness cost is inferred the smaller the relative fitness of response-*on* cells (Fig HII, blue). The homogeneous-response model with constrained mutant fitness (Fig HII, purple) also infers a more severe mutant fitness cost for smaller r_{on} (under both permissive and stressful conditions, since $\rho^p = \rho^s$), but less severe than the unconstrained model version.

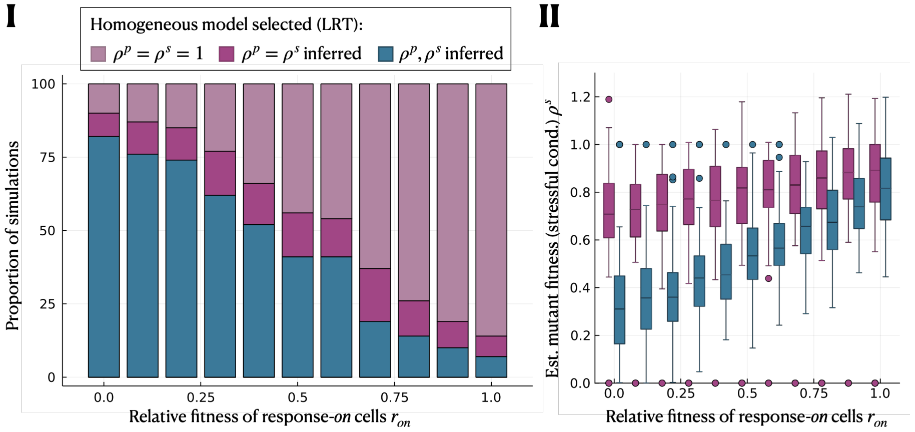

Figure H: **Estimation of mutant fitness cost by the best homogeneous-response model.** Here, we show additional estimation outputs to Results Fig 5. **I** Best homogeneous-response model, as chosen by a likelihood-ratio test in our first step in the model selection procedure. **II** Mutant fitness under stressful conditions as estimated by the homogeneous-response models with constrained (dark purple) or unconstrained (blue) mutant fitness.

J Parameter estimation and model selection for smaller mutation-rate increase

In the model selection in the main Results, we select between heterogeneous and homogeneous responses using simulated data where we set the true mutation-rate increase to $\frac{\mu_{on}}{\mu_{off}} = 100$. Here, we repeat this analysis for a mutation-rate ratio of $\frac{\mu_{on}}{\mu_{off}} = 10$. We find that the number of simulations in which the heterogeneous-response model is selected drops, reaching at most $\sim 25\%$ for small r_{on} (Fig II). In most of the remaining cases, either no model is preferred (more commonly for small r_{on}), or the homogeneous-response model without differential mutant fitness is selected (more commonly for large r_{on}). The homogeneous-response models with inferred mutant fitness are selected in only a few cases. Both heterogeneous and homogeneous-response models remain able to infer the mutation-supply ratio or increase in population mean mutation rate, respectively, reasonably accurately, with only a slight underestimation in both cases (Fig III and IIII).

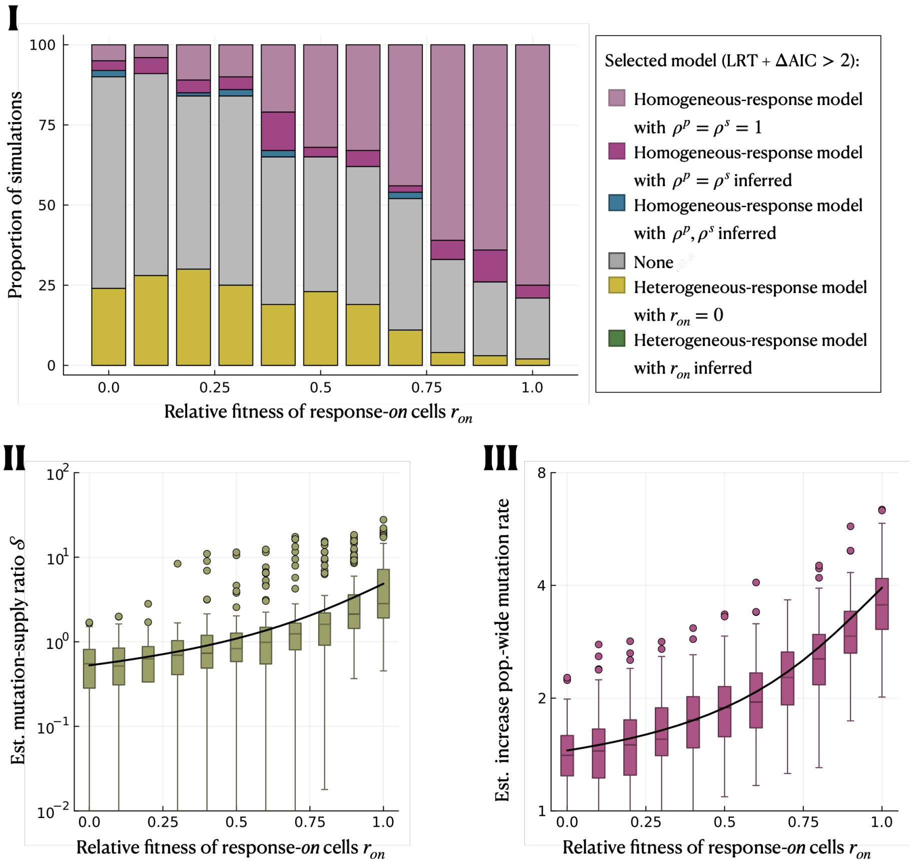

Figure I: **Model selection is more often inconclusive when the mutation-rate increase is smaller.** We simulate using the heterogeneous-response model for a range of relative fitness of response-*on* cells, r_{on}, and with a true mutation-rate ratio of $\frac{\mu_{on}}{\mu_{off}} = 10$; and infer using the same models as considered in the model selection in the main Results. **I** Model selection using LRT and AIC. **II** Estimated mutation-supply ratio, $\mathcal{S}$, by the best heterogeneous-response model. **III** Estimated increase in mutation rate, $\frac{\mu^s}{\mu^p}$, by the best homogeneous-response model. The black lines in **II** and **III** indicate the true values of $\mathcal{S}$ and the increase in population mean mutation rate, $\bar{M}$, respectively. The parameters used in the simulations are $\nu_{on} = 10^{-7}\ h^{-1}$ and $\gamma_{on} \in [0,\ 1]\ h^{-1}$.

K Parameter estimation and model selection when there is no increase in mutation rate

In all results so far, we used simulated data with a true increase in mutation rate under stress ($\mathcal{S} > 0$ for heterogeneous and $\frac{\mu^s}{\mu^p} > 1$ for homogeneous stress responses). Here, we test the parameter estimation and model selection when there is actually no increase in mutation rate under stress. For this purpose, we simulate a homogeneous stress response which does not impact the mutation rate ($\mu^s = \mu^p$), but results in a mutant fitness cost of varying severity ($\rho^p = 1$ and $\rho^s \in [0, 1]$). We then apply our LRT plus AIC two-step model selection procedure (as in the model selection in the main Results). We find that when mutants have a low fitness under stress (small ρ^s), the unconstrained homogeneous-response model is selected in the majority of cases (Fig JI). As the mutant fitness increases, no model is selected for an increasing proportion of simulations, suggesting that both heterogeneous- and homogeneous-response models can explain the data similarly well. Regardless of which model is selected, both perform fairly well in inferring little or no increase in mutation rate. In the heterogeneous-response model (Fig JII), a mutation-supply ratio of $\mathcal{S} = 0$ corresponds to no contribution from response-*on* cells, and the estimated $\mathcal{S}$ tends towards zero as mutant fitness cost decreases. For small mutant fitness, $\mathcal{S}$ is slightly over-estimated (median estimate $\mathcal{S} \approx 0.5$ for $\rho^s < 0.5$). This result again reflects the similarity in mutant count distributions produced by either the heterogeneous-response model with non-dividing response-*on* cells ($r_{on} = 0$) or the homogeneous-response model with severe mutant fitness cost (small ρ^s), cf. Fig B. Under the homogeneous-response model (Fig JIII), the estimated mutation-rate increase $\frac{\mu^s}{\mu^p}$ is unbiased, centred around the true value of 1, regardless of mutant fitness cost.

L Parameter estimation and model selection for different switching rates

In the model selection in the main Results, we perform model selection on simulated mutant count data from the heterogeneous-response model with varying relative fitness of response-*on* cells. Here, we carry out a similar analysis on simulations with varying relative switching rates $\tilde{\alpha}$ (i.e. rate of switching the response *on* under stress). Specifically, we simulate under the heterogeneous-response model without cell death and with non-dividing response-*on* cells ($r_{on} = 0$), and perform model selection between heterogeneous and homogeneous-response models using LRT plus AIC.

When the relative switching rate is small ($\tilde{\alpha} < 0.01$), the mutation-supply ratio $\mathcal{S} \ll 1$, which implies very little increase in population mean mutation rate under stressful conditions. In this case, we find that no model is selected in the majority of simulations (Fig KI), which is in accordance with the results when there is actually no increase in mutation rate

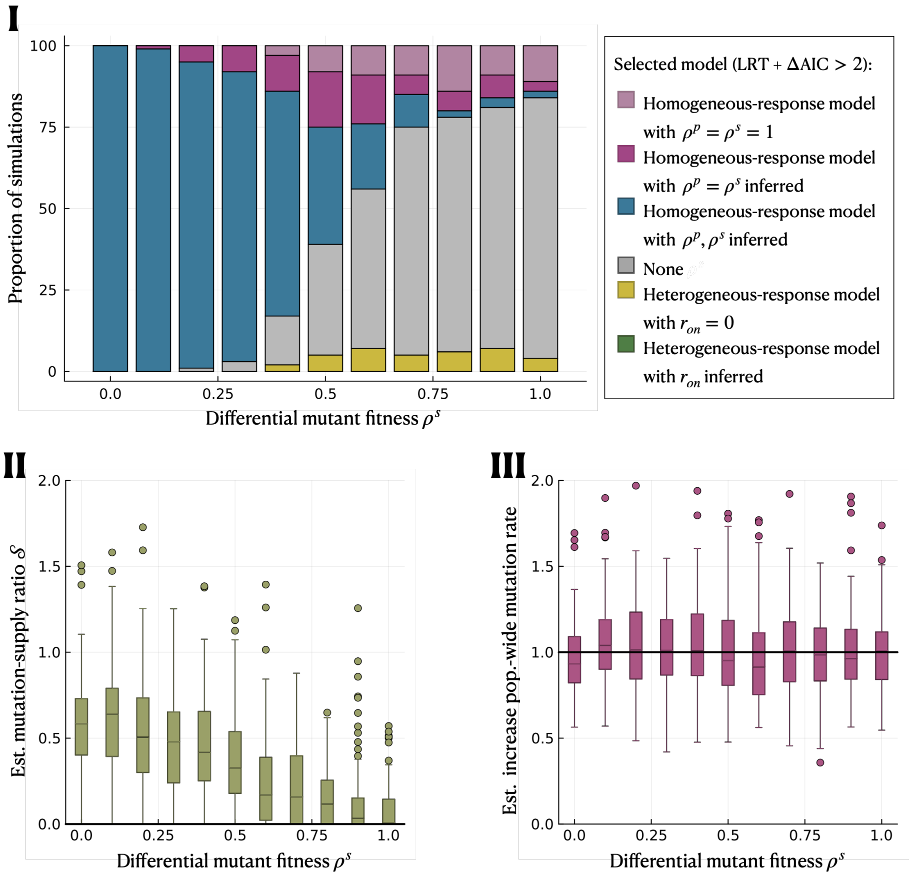

Figure J: **Model selection when there is no increase in mutation rate under stress.** We simulate under the unconstrained homogeneous-response model without an increase in mutation rate ($\mu^s = \mu^p$). **I** Model selection using the LRT and AIC. **II** Estimated mutation-supply ratio, $\mathcal{S}$, by the best heterogeneous-response model. **III** Estimated increase in mutation rate, $\frac{\mu^s}{\mu^p}$, by the best homogeneous-response model. The black lines in **II** and **III** indicate the true values ($\mathcal{S} = 0$ and $\frac{\mu^s}{\mu^p} = 1$, respectively). The parameters used in the simulations are $\rho^p = 1$ and $\rho^s \in [0,\ 1]$.

(previous section). For a relative switching rate of $\tilde{\alpha} = 0.05$, the heterogeneous-response model is selected in slightly less than 75% of simulations, similarly to our results with $r_{on} = 0$ in Results Fig 5, where we simulated under identical parameters. Interestingly, however, the fraction of simulations in which the heterogeneous-response model is selected does not increase further for larger $\tilde{\alpha}$. This implies that even for large relative switching rates, heterogeneous stress responses or mutant fitness cost under stress represent alternative model explanations for similar patterns in mutant count distributions and cannot be distinguished from fluctuation assay data alone.

The estimation of the mutation-supply ratio $\mathcal{S}$ by the best heterogeneous-response model is accurate and precise for sufficiently large relative switching rate $\tilde{\alpha}$ (Fig KII), which implies large $\mathcal{S}$, in accordance with our results presented in Results Fig 2. Furthermore, the increase in mutation rate estimated by the best homogeneous-response model ($\frac{\mu^s}{\mu^p}$) is a fairly accurate and precise estimate of the true increase in population mean mutation rate ($\bar{M}$) in the heterogeneous-response simulations, with only a slight underestimation of $\bar{M}$ for intermediate $\tilde{\alpha}$ (Fig KIII).

M Model selection limited to constrained mutant fitness in the homogeneous-response case

In last main Results section, we simulate under the heterogeneous-response model for a range of relative fitness of response-*on* cells (r_{on}) and perform model selection between the homogeneous-response model (a) without differential mutant fitness (setting $\rho^p = \rho^s = 1$), (b) with constrained differential mutant fitness, i.e. one additional inference parameter $\rho^s = \rho^s$, or (c) with unconstrained differential mutant fitness, i.e. two additional inference parameters ρ^s and ρ^s; and the heterogeneous-response model (d) with zero fitness of response-*on* cells (setting $r_{on} = 0$), or (e) with r_{on} and f_{on} as two additional inference parameters. Moreover, we evaluate the homogeneous-response models' performance in estimating the true increase in population mean mutation rate ($\bar{M}$) and the heterogeneous models' performance in estimating the true mutation-supply ratio ($\mathcal{S}$). Here, we repeat this analysis, but we consider only the homogeneous-response models (a) and (b), in which the mutant fitness under stressful and permissive conditions is equal ($\rho^p = \rho^s$). This represents a reasonable starting assumption in the absence of any *a priori* reason to expect that mutant fitness costs should differ in these conditions.

Performing model selection using LRT plus AIC, we find that the heterogeneous-response model with $r_{on} = 0$ is selected in most cases when the true relative fitness of response-*on* cells (r_{on}) is small (Fig LI). For intermediate values of r_{on}, the heterogeneous-response model with r_{on} inferred is selected more often (up to $\sim 30\%$), but no model is selected even more often (up to $\sim 40\%$). For large values of r_{on}, the homogeneous-response model

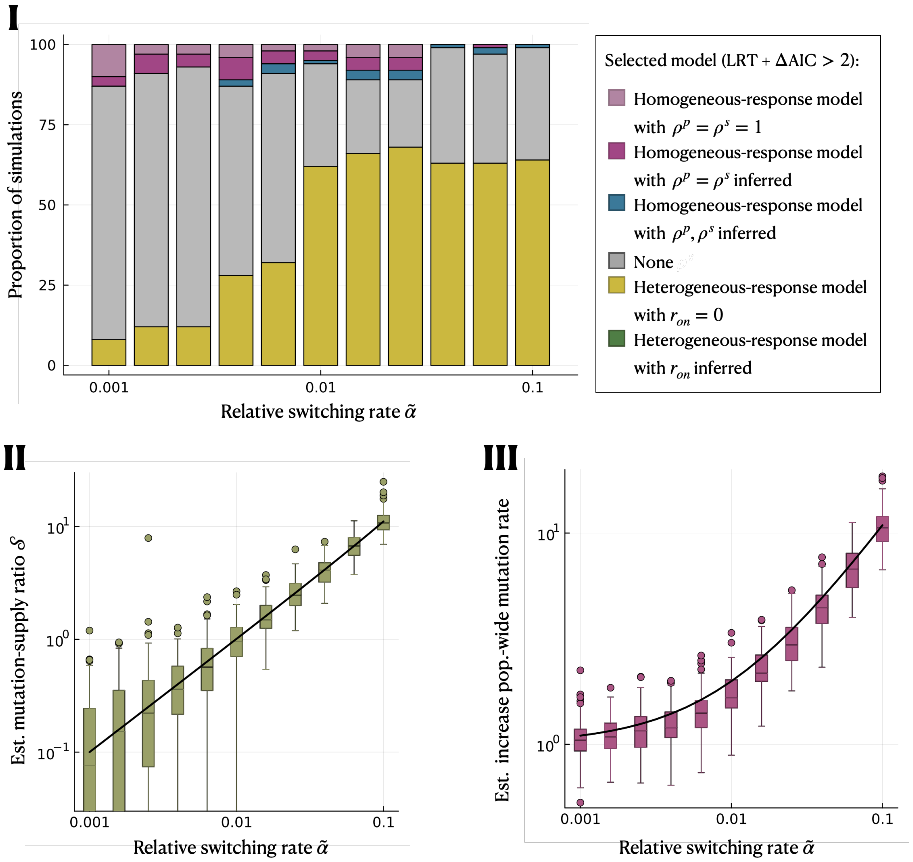

Figure K: **Model selection for varying relative switching rates.** We simulate under the heterogeneous-response model for a range of relative switching rates, $\tilde{\alpha}$. **I** Model selection between heterogeneous and homogeneous stress response models using LRT and AIC. **II** Estimated mutation-supply ratio, $\mathcal{S}$, by the best heterogeneous-response model. **III** Estimated increase in mutation rate, $\frac{\mu^s}{\mu^p}$, by the best homogeneous-response model. The black lines in **II** and **III** indicate the true values of $\mathcal{S}$ and the increase in population mean mutation rate, $\bar{M}$, respectively. The parameter range used in the simulations is $\alpha \in [0.001,\ 0.1]\ h^{-1}$.

without differential mutant fitness ($\rho^s = \rho^p = 1$) is selected in the majority of simulations. The homogeneous-response model with constrained differential mutant fitness is selected in only a few simulations, with the highest percentage of $\sim 10\%$ for intermediate r_{on}.

Overall, over the whole parameter range, the heterogeneous-response model is selected more often when we constrain mutant fitness to be equal under stressful and permissive conditions (Fig LI) compared to when it is also allowed to be unconstrained (Results Fig 5). The differential mutant fitness itself ($\rho^p = \rho^s$) is estimated to be around $\rho^s \approx 0.65$ over the whole range of r_{on} (Fig LII). Moreover, we find that constraining the mutant fitness results in a slight underestimation of the increase in population mean mutation rate $\bar{M}$, especially for small r_{on} (Fig LIII).

N Comparing model selection procedures

In the model selection in the main Results, we use a two-step procedure involving (i) a likelihood-ratio test (LRT) to choose the best homogeneous and the best heterogeneous model version, then (ii) the AIC to compare these two best models. There, we simulated under the heterogeneous-response model with $c = 50$ parallel cultures per fluctuation assay. Here, we analyse the performance of our model selection procedure for smaller numbers of parallel cultures ($c = 20$, 10). We also assess model selection when simulating under the homogeneous-response model. Moreover, in all cases, we show how results using the AIC in the second selection step compare to using the Bayesian information criterion (BIC). The BIC is defined as

$$\text{BIC} = k \ln n - 2 \ln \mathcal{L} \tag{8}$$

with k being the number of inferred parameters and $n = 2c$ the number of data points, i.e. the total number of parallel cultures simulated under permissive plus stressful conditions. As for the AIC, we say that the BICs of two models are comparable if their difference is within ± 2. For an overview of the advantages and disadvantages of different model selection techniques, including AIC and BIC, see [3].

First, we evaluate the performance of the above-described model selection procedures when simulating under the heterogeneous-response model (Fig M) as done in Results Fig 5. When using $c = 50$ parallel cultures in the inference, the model selection procedure using BIC instead of AIC is more conclusive: BIC more often selects the heterogeneous-response model at small r_{on} and the homogeneous-response model with no differential mutant fitness as large r_{on}, whereas AIC selects no model in a larger fraction of cases. Interestingly, when fewer parallel cultures are used in the inference ($c = 20$, 10), the AIC selects the heterogeneous-response model less often when r_{on} is small but more often when r_{on} is large. Model selection using BIC, on the other hand, is more often inconclusive when using fewer parallel cultures, and performs similarly to model selection using AIC when $c = 10$.

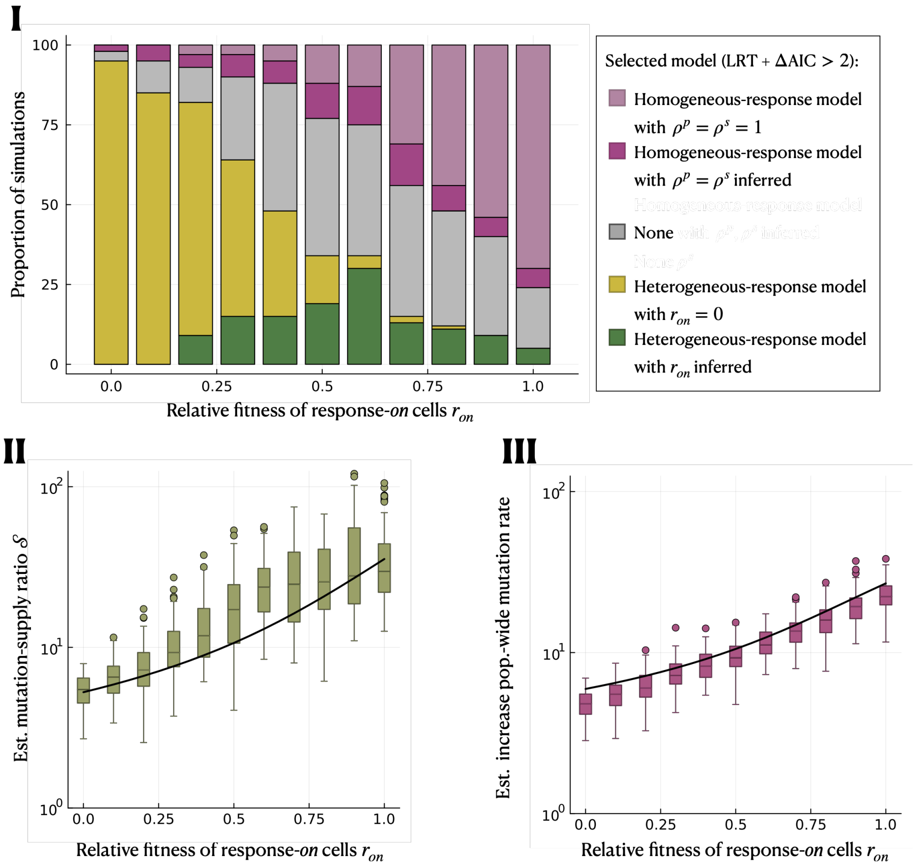

Figure L: **Model selection with constrained mutant fitness.** We use the same simulated data as in Results Fig 5, but for inference, we exclude the version of the homogeneous-response model with unconstrained mutant fitness. **I** Model selection amongst homogeneous response models with constrained mutant fitness (a-b) and heterogeneous-response models (d-e) using LRT and AIC. **II** Estimated mutation-supply ratio, $\mathcal{S}$, by the best heterogeneous-response model. **III** Estimated increase in mutation rate, $\frac{\mu^s}{\mu^p}$, by the best homogeneous-response model (a) or (b). The black lines in **II** and **III** indicate the true values of $\mathcal{S}$ and the increase in population mean mutation rate, $\bar{M}$, respectively. The parameter range used in the simulations is $\gamma_{on} \in [0, 1]\ h^{-1}$.

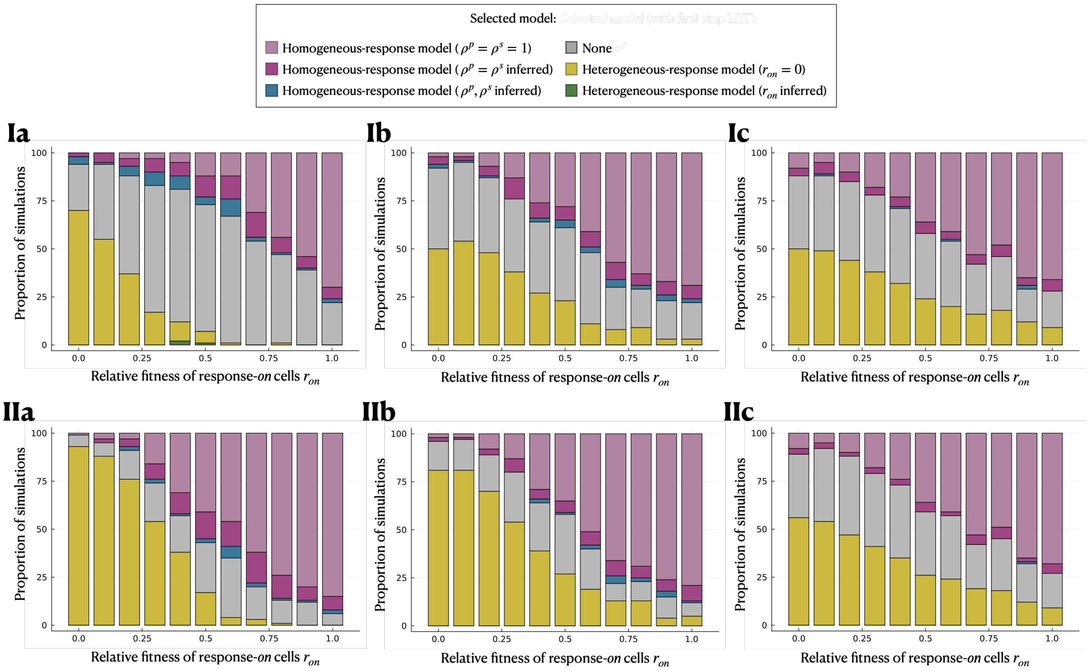

Figure M: **Model selection when simulating under the heterogeneous-response model.** We use the same simulation parameters as in Results Fig 5 and use a two-step model selection procedure with LRT as the first step and either AIC (**I**) or BIC (**II**) as the second selection step, and $c = 50$ (**a**), $c = 20$ (**b**) or $c = 10$ (**c**) parallel cultures in the inference.

The latter is expected as for $c = 10$ the penalising constant used in the BIC (Eq 8) equals $\ln 2c \approx 3$, close to the penalising constant (= 2) used in the AIC.

Next, we perform model selection when simulating under the homogeneous-response model with constrained mutant fitness cost ($\rho^p = \rho^s \in [0, 1]$), for various increases in population-wide mutation rate (Fig N). Again, we compare the performance of model selection procedures using AIC (**I**) or BIC (**II**). In all cases, the rate of false positives, i.e. selecting the heterogeneous-response model, is low; up to maximally $\approx 10\%$ for mutant fitness costs around $\rho^p = \rho^s = 0.8$.

Finally, we perform model selection when simulating under the homogeneous-response model with differential mutant fitness only under stressful conditions ($\rho^p = 1$ and $\rho^s \in [0, 1]$) and various increases population-wide in mutation rate (Fig O), using the AIC (**I**) or BIC (**II**) for model selection. For less severe mutant fitness costs ($\rho^s \to 1$), AIC tends to select the homogeneous-response model without differential mutant fitness regardless of

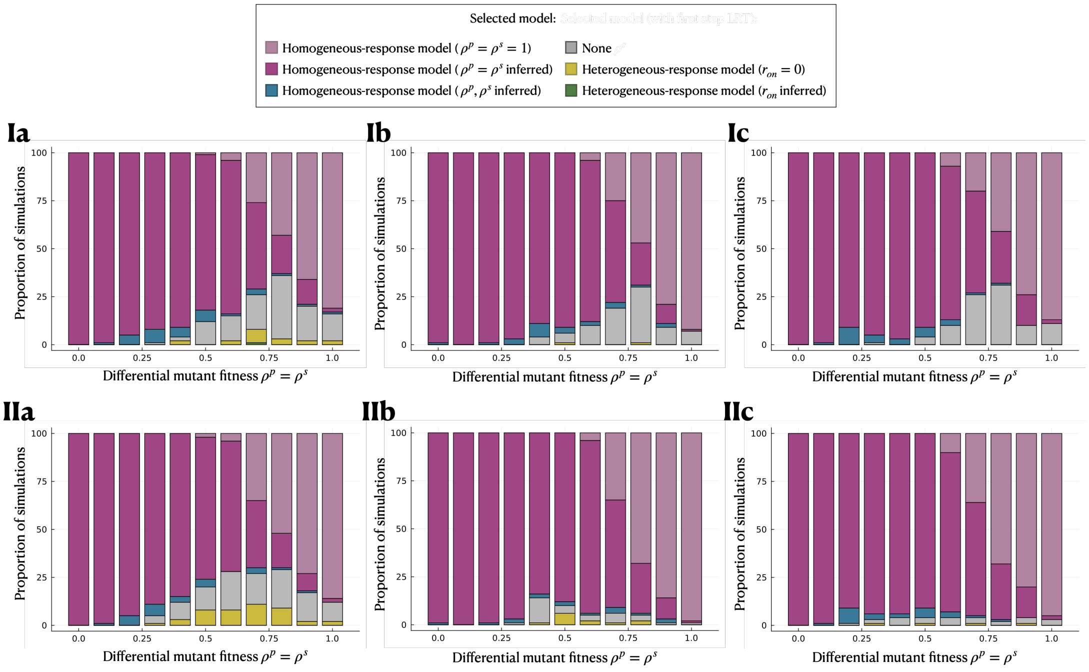

Figure N: **Model selection when simulating under the homogeneous-response model with constrained mutant fitness.** We use a two-step model selection procedure with LRT as the first step and either AIC (**I**) or BIC (**II**) as the second selection step, and a simulated increase in mutation rate of $\frac{\mu^s}{\mu^p} = 3.2$ (**a**), $\frac{\mu^s}{\mu^p} = 10$ (**b**) or $\frac{\mu^s}{\mu^p} = 32$ (**c**). The parameter range used in the simulations is $\rho^p = \rho^s \in [0,\ 1]$.

the true mutation-rate increase. For a severe mutant fitness cost (small ρ^s), the model selected by AIC depends on the true increase in mutation rate. When the mutation-rate increase is small ($\frac{\mu^s}{\mu^p} = 3.2$, left column), the AIC usually correctly selects the unconstrained homogeneous-response model. As the mutation-rate increase becomes larger, the model selection first becomes frequently inconclusive (for $\frac{\mu^s}{\mu^p} = 10$, middle column) and then tends to select the heterogeneous-response model with $r_{on} = 0$ (for $\frac{\mu^s}{\mu^p} = 32$, right column). The reason for this behaviour is that the underlying mutant count distributions are similar for both the homogeneous-response model with a large mutant fitness cost and the heterogeneous-response model with non-dividing response-*on* cells (recall Fig B), and the latter model has fewer inferred parameters.

In contrast, model selection using the BIC (Fig O, bottom) has a high rate of false positives, i.e. selecting the heterogeneous-response model (with $r_{on} = 0$) even though we simulated under the homogeneous-response model. For smaller mutation-rate increases ($\frac{\mu^s}{\mu^p} = 3.2$ and $\frac{\mu^s}{\mu^p} = 10$), the false-positive rate is highest when the mutant fitness cost is small, whereas for larger mutation-rate increase ($\frac{\mu^s}{\mu^p} = 32$), the false-positive rate is highest at intermediate ρ^s. For sufficiently small mutant fitness cost ($\rho^s \to 1$), BIC usually selects the homogeneous-response model with no mutant fitness cost ($\rho^s = \rho^p = 1$) regardless of the true mutation-rate increase.

To summarise, model selection using the BIC is more conclusive, with a higher rate of true positives (i.e. correctly selecting the heterogeneous-response model when it was used for simulations) than model selection using the AIC (Fig M). Both AIC and BIC show a low rate of false positives (i.e. incorrectly selecting the heterogeneous-response model) when simulating under the homogeneous-response model with equal mutant fitness costs under permissive and stressful conditions (Fig N) or a sufficiently small mutant fitness cost under stressful conditions only (Fig O). However, using the BIC results in a higher rate of false positives when simulating under the homogeneous-response model where mutants have a sufficiently large fitness cost only under stressful conditions (Fig O). For this reason, we decided to use AIC as our model selection procedure when presenting the main results. However, in the file called **inference.jl** at `https://github.com/LucyL-J/Quantifying-SIM`, we provide code to use either AIC or BIC in model selection.

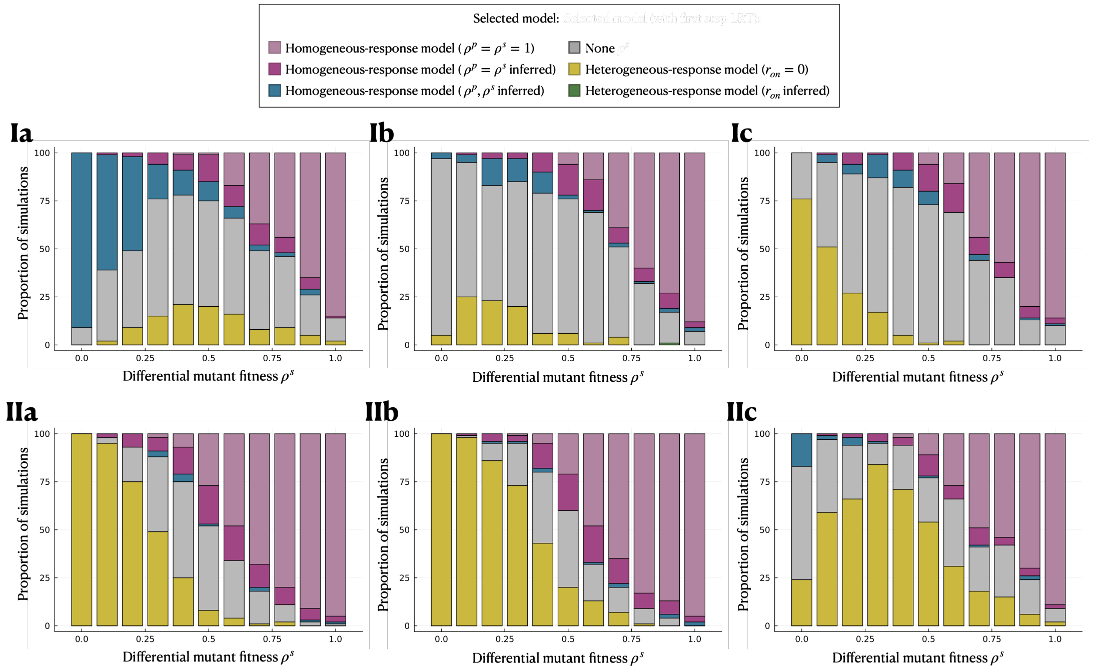

Figure O: **Model selection when simulating under the homogeneous-response model with mutant cost only under stressful conditions.** We use a two-step model selection procedure with LRT as the first step and either AIC (**I**) or BIC (**II**) as the second selection step, and a simulated increase in mutation rate of $\frac{\mu^s}{\mu^p} = 3.2$ (**a**), $\frac{\mu^s}{\mu^p} = 10$ (**b**) or $\frac{\mu^s}{\mu^p} = 32$ (**c**). The parameter range used in the simulations is $\rho^p = 1$ and $\rho^s \in [0,\ 1]$.

References

[1] Frenoy A, Bonhoeffer S. Death and population dynamics affect mutation rate estimates and evolvability under stress in bacteria. PLOS Biology. 2018 may;16(5):e2005056. Available from: https://dx.plos.org/10.1371/journal.pbio.2005056.

[2] Zheng Q. Estimation of Rates of Non-neutral Mutations When Bacteria are Exposed to Subinhibitory Levels of Antibiotics. Bulletin of Mathematical Biology. 2022 nov;84(11):131. Available from: https://doi.org/10.1007/s11538-022-01085-5https://link.springer.com/10.1007/s11538-022-01085-5.

[3] Ding J, Tarokh V, Yang Y. Model Selection Techniques: An Overview. IEEE Signal Processing Magazine. 2018;35(6):16-34.
