# Supplementary material for: Estimating mutation rates under heterogeneous stress responses
Source: PLoS Comput Biol. 2024 May 28;20(5):e1012146. doi: 10.1371/journal.pcbi.1012146 (PMC11161091; doi:10.1371/journal.pcbi.1012146)
Supplement: Attachment — Submitted filename: response.docx [file pcbi.1012146.s002.docx]

# **Response to reviews**

# We thank the editors and reviewers for their detailed reading of our manuscript and insightful comments. We are pleased that both reviewers recognised the novelty and importance of our work, and we are confident that thoroughly addressing their comments has greatly strengthened the manuscript.

# The editors highlighted the following four major criticisms. Here, we briefly summarise how we addressed each of these key issues and follow up with a detailed point-by-point response to the reviewers below.

1. **Alternative model selection methods:** We have extensively explored alternative model selection methods and evaluated their performance. On this basis, we decided to adjust our previous approach, which used only AIC for model selection. Now, we have implemented a new two-step model selection procedure. In the first step, we use a likelihood ratio test (LRT) to select the best of a set of nested homogeneous models and, separately, to select the best of a set of nested heterogeneous models. In the second step, we use the AIC to select between the best homogeneous and the best heterogeneous model. (For this selection, we cannot use the LRT, as none of the homogeneous models is nested within any of the heterogeneous ones.) We have additionally tested the BIC (which we show in the SI section S14) and cross-validation (results provided in the response to the reviewer) as alternative selection criteria for the second step, but have found the AIC to perform best overall.
2. **Application to empirical data:** We agree that our method could, in principle, be applied to experimental data, and we have developed our methods with this eventual aim in mind. The minimal requirement for suitable experimental data is to carry out fluctuation assays in both permissive and stressful conditions and provide the raw mutant count data. Ideally, if the stress response was known to be heterogeneous and the fraction of response-on cells (f_on_) had been estimated under the same conditions, we expect our model to perform well in estimating the mutation rate increase (cf. Results section 3.1). However, unfortunately we are not aware of any study to date that has estimated the fraction of response-on cells *and* carried out fluctuation assays in parallel. Alternatively, in the absence of a measurement of f_on_, we could still attempt to detect heterogeneity from fluctuation assay data alone through our model selection procedures (cf. Results section 3.4). However, as we have shown here with simulated data, through large areas of parameter space, it is difficult to distinguish between alternative models that explain mutant count data similarly well. We are aware of a few published experimental datasets that would meet the minimal requirements of reporting fluctuation assays in both permissive and stressful conditions, but we see limited value in analysing a single case study, given the difficulties in model identifiability. We believe a meta-analysis of multiple experimental studies would have much greater value, but this is unfortunately beyond the scope of our current paper, and we, therefore, aim to address it in our future work.
3. **Confidence intervals on parameter estimates:** We have now implemented a method of calculating profile likelihood confidence intervals (CIs) on all parameter estimates, and included this method in our deposited code. We describe the method in Model & Methods section 2.4.2 and provide results figures in SI (Figure S4).
4. **Organisation of manuscript:** To improve readability, we extensively restructured the Model & Methods section and addressed all of the reviewers’ specific points. In the new version, we first recap what we call the “standard model” of classical fluctuation assays with relevant extensions (section 2.1); then, we formalise the homogeneous model implicitly used in studies of SIM to date and set out our notation (section 2.2), followed by a detailed description of our new heterogeneous model (section 2.3). We have also updated our model schematic (Figure 1) for greater clarity and refer back to this figure throughout the manuscript for additional clarity.

**Reviewer #1:**

The manuscript by Lansch-Justen et al. describes theory and modelling approaches to estimate mutation rates in bacteria. The work is novel and important because it addresses a crucial gap in the theoretical tools available to model and quantify mutagenesis. Specifically, existing methods to estimate mutation rates assume that all cells in a population have the same mutation rate. However, recent experimental studies have shown that this is not the case, especially under stress conditions when mutation rates increase heterogeneously in a bacterial population. Fluctuation tests are the most common technique to estimate mutation rates in the lab. The method is seemingly straightforward but quantification of the data is mathematically surprisingly complex. For example, it is unclear whether the mutant count distribution obtained from a fluctuation test can tell whether mutation rates vary between cells, and how reliably various parameters can be estimated from the measured data. This work describes a simple model where a population of bacteria splits into two subpopulations with different mutation rates, fitness, and viability in response to stress. Equations are derived to estimate these parameters from data of simulated fluctuation tests. The results show under what conditions model inference and parameter estimation are reliable and when they are not. This provides a useful theoretical footing for the design of future experimental studies.

We thank the reviewer for these positive comments and excellent summary of our study.

I suggest addressing the following minor points before publication:

1. Abstract: “Moreover, we find that in many cases, our model of heterogeneous stress responses and the standard model with mutant fitness cost reproduce fluctuation assay data equally well, suggesting that separate experiments would be required to identify the true underlying process.” I suspect that many readers would struggle to understand this sentence since it’s not necessarily well known what “the standard model with mutant fitness cost” is. I found the Author Summary more informative than the Abstract. In the Abstract, we have rephrased this sentence to avoid the term “standard model”.
2. Line 53: specify error-prone “DNA” polymerases We added the term “DNA”.
3. Line 90 following: “Single-cell studies using fluorescent reporters for the SOS response in E. coli have revealed that its expression is highly heterogeneous and that this heterogeneity can be approximated as a bimodal response, with some cells expressing low levels of SOS under DNA damage whilst others show a very high level of expression”. It is debatable whether SOS induction is bimodal; studies that reported distributions of SOS expression levels showed a unimodal population with a tail rather than bimodal populations. The discrete on/off model is a reasonable simplification for the purpose of this study (to keep it mathematically tractable), but it should be acknowledged that it doesn’t match the experimental data precisely. We agree with the referee that depending on studies, the SOS response can be shown as unimodal with a tail (see, for example, <https://pubmed.ncbi.nlm.nih.gov/30948267/> ) or bimodal (see, for example, <https://pubmed.ncbi.nlm.nih.gov/35620827/> ), which may be linked to various types of DNA damage and differences between long-term exposure to low level of damage versus short-term exposure to higher level. To avoid confusion, we now write (Introduction Line 97): “Under certain conditions, a subpopulation of cells with a very high level of SOS compared to the rest of cells with lower expression levels has been observed, and this heterogeneity can be approximated as a bimodal response”. We also return to this point in the Discussion (Line 816).
4. Effect of stress response on cell division rate: The SOS response is known to inhibit cell division while cell growth continues, leading to cell filamentation. This should be mentioned in the manuscript and the effects on mutation rate estimate discussed (especially since the authors have studied this effect before [https://pubmed.ncbi.nlm.nih.gov/2947](https://pubmed.ncbi.nlm.nih.gov/29470493/)0493/). Yes, the role of filamentation is a very relevant consideration and we apologise that we did not clearly explain our thinking as to how it impacts the production of mutants. We now address this point in the model description (section 2.3.1, paragraph “Mutant population dynamics”, Line 271ff). Filamenting cells continue to replicate their chromosomes, and mutations are linked to chromosome replication, not directly to cell division. The precise dynamics of these multinucleated cells, particularly the fate of mutant chromosomes, is not very well understood, especially when a low-level stressor is maintained over a time period of many hours, as in the growth phase of a fluctuation assay. The most relevant study of which we are aware is by Bos et al. 2015 (https://pubmed.ncbi.nlm.nih.gov/25492931/), which we now cite to support our model assumption. There, the authors observed budding of normal-sized cells primarily from the tips of the filaments, of which a substantial fraction are viable and could contain mutated chromosomes. Although our model remains a simplification of this process, overall this evidence indicates it is reasonable to assume that a non-dividing filament can produce mutant offspring. The ‘mutation rate’ should be interpreted as an effective rate at which mutant chromosomes arise and are segregated into a mutant cell, which we also now clarify in the text. The reference the reviewer mentions (Sun et al. 2018) deals with a different situation leading to cells having multiple copies of chromosomes: conditions of rapid growth inducing multi-fork replication rather than cell filamentation under stress. We expect the modes of chromosome segregation and cell division to be different and, therefore, do not directly build on or cite the work of Sun et al. However, we agree this would be an interesting avenue to explore in future work.
5. The model considers the case where cells switch from the response-off to response-on state during stress. It would be useful to discuss to what extent the results can be extrapolated to situations where cells switch back to the response-off state (relevant for e.g. recovery after stress removal, or stochastic switching between on and off states during unstressed conditions or low stress levels). The reviewer is correct that stress response regulation can be more complex than we considered here. In our model, we assume that cells that switch *on* the response do not switch it back *off* during the growth phase since the stressor remains present, but that once plated out on the selective plates where the stressor is not present, all response-*on* mutants resume dividing enough to form a visible colony. We state these assumptions in the model description (section 2.3.1, paragraph “Mutant population dynamics”, Line 271ff). We neglect any stochastic switching during unstressed (permissive) conditions, which is reasonable because we expect the subpopulation of response-on cells to be very small in these conditions and, thus, to make a negligible contribution to mutations (i.e. the mutation-supply ratio, now called S in the manuscript, is <<1). However, motivated by your comment, we have updated our Discussion to better address the scenario of stochastic switching in absence of a stressor. We now clarify that our method is insensitive to low rates of switching *on* the response (Line 824).

6. “Section 2.2.1 Inferred and measured parameters” was a bit confusing to read because it repeats almost the same text for the heterogeneous and homogeneous models. This could be improved by describing the common parameters first, and then noting the differences between the two models? To improve clarity, we have substantially revised this section (now appearing as section 2.4.1: Parameters in the inference). We separated this section into two paragraphs with headings “Homogeneous-response model:” and “Heterogeneous-response model:” for a clearer structure, and added a table (Table 1) listing all parameters in the inference, which indicates which model(s) each parameter appears in.
7. Line 405: “For each parameter combination, we simulate R = 100 pairs of fluctuation assays under permissive and stressful conditions, with c = 50 parallel cultures per assay.” How sensitive are the parameter estimation and model inference results described here to the number of assays and cultures per assay? Can this be added as a supplementary figure? It would provide useful guidance for the design of experiments. Also, how do these values compare to what is typically done in the lab? It would be good to cite some papers here to justify the chosen values. The number of simulated assays (R=100) is arbitrary, simply chosen to get a reasonable overview of the range of variation amongst simulations. Parameter estimation and model selection are applied to each simulated assay individually. The value of c=50 parallel cultures per assay is on the high side of what experimenters typically use but is perfectly feasible with culturing on a 96-well plate. We now justify this choice in section 2.5.1 (Simulation Methods, Line 467) with reference to an experimental protocol (<https://pubmed.ncbi.nlm.nih.gov/31840662/>). However, experiments are often made with fewer parallel cultures, and we agree with the reviewer that the sensitivity of our results to c is relevant. We have tested the sensitivity of Results section 3.1 to the number of parallel cultures c, see SI Figure S4, and repeated the analysis in Results section 3.4 with smaller numbers of parallel cultures (c=20,10), which we provide in SI Figure S13.
8. Line 412: “beginning with the simplest model of heterogeneous stress responses without cell death, with mutant fitness equal to non-mutant fitness, and with zero division rate of response-on cells.” I was wondering why the authors didn’t start with the simpler model of homogenous stress response for simplicity, and subsequently move on to the more complex heterogeneous stress response case? In the Model & Methods section, we now follow the reviewer’s suggestion and start by briefly describing what we call the “standard model” (section 2.1), followed by the homogeneous-response model already widely used to describe stress responses (section 2.2), and finally, describe in more detail our novel heterogeneous-response model (section 2.3). In the Results section 3.1 (which the reviewer’s comment directly refers to), we do not test the ability of the standard or homogeneous model to infer parameters, as this is already well established. Instead, we test only the performance of our new method for heterogeneous responses. We bring up the homogeneous model again in the context of model selection (Results section 3.4).
9. Line 413: The case of zero division rate of response-on cells seems rather extreme. If the response-on cells do not divide at all, then shouldn’t this cancel much of the increase in their mutation rate? I probably missed something here, but it could be helpful to explain this point more clearly. Zero division rate is indeed the extreme lower bound but it is not unrealistic (to a first approximation) for cells inducing the SOS response, our main inspiration. The reviewer is correct that, even though response-on cells produce more *mutations* per capita*,* each of these mutations results only in a single *mutant cell* if response-on cells are non-dividing. This implies, overall, a potentially small total mutant count, but crucially, a change in the shape of the mutant count distribution compared to the standard model (from heavy-tailed towards Poissonian), which is, in fact, crucial to disentangle the contributions to the total mutant count from the response-on and off subpopulations in the inference. In the new version of the manuscript, we introduce the concept of mutation-supply ratio early on (Model & Methods, Line 324) to build intuition about the relative contribution of the response-on subpopulation. We have also added example mutant count distribution plots, illustrating this shift in shape (SI Figure S2).
10. It would be useful to include a figure before figure 2 with representative model outputs that show the number of cells in the response-on and response-off populations vs time along with the number of mutants vs time. This could be shown for a few different parameter sets and model types (homogenous, heterogeneous, with/without fitness differences, with/without death, etc). This should give the reader a better intuition for the more abstract results about the inference method performance. Thank you for this suggestion. However, after trying out some figure versions, we felt that showing model outputs over time is difficult to interpret because (i) individual simulation realisations show great variation and (ii) in a fluctuation assay, only a snapshot in time (at the end of the growth phase) is captured anyway. Instead, to help build intuition, we added Figure S2 in the SI with example mutant count distributions arising from the homogeneous and the heterogeneous-response models for a few parameter choices that we think best illustrate our point about the shift in the shape of the distribution (see response to comment 9).
11. Another relevant paper to cite: <https://pubmed.ncbi.nlm.nih.gov/30409883/> This paper is very interesting but focuses on heterogeneity in the expression of efflux pumps rather than in a stress response. Since we limit our scope to stress response, we decided not to cite it in this context.
12. Note that the situation for alkylation stress response in reference 20 actually matches the described model quite well, but in the opposite way. Here, the response-off population has a higher mutation rate and lower survival than the response-on population. To what extent can the theoretical findings be applied to this opposite case? Thank you for this insightful comment. We agree that it would indeed be possible to apply the same model structure (coupled ODEs for the non-mutant population dynamics, two-type branching process for the mutant dynamics) to the case of the alkylation stress response as described by the reviewer. We could capture the key features of this response with two states: (1) high mutation rate and high death rate (associated with response off) and (2) low mutation rate and low death rate (associated with response on), with all cells initially in state (1) and with unidirectional switching from (1) 🡪 (2). Note that the parameters associated with state 2 (response on) would now be (to a reasonable approximation) equal to permissive conditions. Because of these differences in parameterisation, unfortunately, we could not directly apply our present findings to this case. Firstly, the approximations we make to derive the mutant count distribution would no longer be valid for the alkylation stress response, in particular, the assumption that response-on cells divide much slower than response-off cells. The difference in death rates would also be crucial to take into account. Secondly, the equivalence of parameters in response-on to permissive conditions would change the joint parameter inference settings. We now included a brief version of this explanation in the Discussion (Line 842).

**Reviewer #2:**

I have read the manuscript titled “Estimating mutation rates under heterogeneous stress responses”.
The authors developed a population dynamic model and maximum likelihood approach to estimate mutation rate heterogeneity from fluctuation assays. This is an important first step towards this goal: evidence for mutation rate heterogeneity (i.e., phenotypic plasticity), especially under stress (González et al 2008) but even without it (Uphoff et al 2016), has been accumulating. I therefore salute the authors for this first step.

• I have some issues that I think the authors can help resolve. Some are related to the presentation– I think the methods section can be organized better and I think some of the methods are not explicitly explained, although I might have missed the explanation (which leads back to better organization…).
• I think the model selection section parts might be enhanced is other methods (CV or LRT) are used.
• I wonder if the authors could have applied their method on some previously published dataset (many fluctuation assays have been published!) and thus demonstrate its usefulness on real data. I appreciate that this might not be feasible because of the requirements of this new method, but in that case I would like to read a clear explanation of what kind of experimental setup is required to use the new method.
• Finally, I think the authors should consider if their method can produce some kind of confidence intervals around the maximum likelihood point estimates; biologists usually want to see something like that when they estimate continuous quantities.

More details on these comments, and more comments, including very minor ones, are listed below.

Thank you for the positive summary along with excellent suggestions for improvement. We have summarised our responses to the four main issues you mentioned above in our response to the editor at the start of this document. We provide point-by-point replies to your detailed comments below.

**Major comments:**
1. Have you considered applying your method on available data? Maybe even the data from the original Luria-Delbrück paper? See for example Holmes et al, Physical Biology 2017 <https://doi.org/10.1088/1478-3975/aa8230>. It could be interesting to see (1) if your method performs well on real data; and (2) if your method is able to detect heterogeneity in data for which no heterogeneity has been previously reported. We agree that it would be interesting to apply our method to experimental data, and we have developed our methods with this eventual aim in mind. The minimal requirement for suitable experimental data is to carry out fluctuation assays in both permissive and stressful conditions and provide the raw mutant count data, which is not the case for most studies using fluctuation assays to estimate mutation rates (including the original Luria-Delbrück paper), unfortunately. Ideally, if the stress response was known to be heterogeneous and the fraction of response-on cells (f_on) had been estimated under the same conditions, we could test whether our model performs well in estimating the mutation rate increase. However, unfortunately we are not aware of any study to date that has estimated the fraction of response-on cells *and* carried out fluctuation assays in parallel. Alternatively, in the absence of a measurement of f_on, we could still attempt to detect heterogeneity from fluctuation assay data alone through our model selection procedures. However, as we have shown here with simulated data, through large areas of parameter space, it is difficult to distinguish between alternative models that explain mutant count data similarly well. We are aware of a few published experimental datasets that would meet the minimal requirements of reporting fluctuation assays in both permissive and stressful conditions, and these would provide interesting test cases for your second question; to see if our method can detect heterogeneity where it was not already reported. However, we see limited value in analysing a single case study, given the difficulties in model identifiability. We believe a meta-analysis of multiple experimental studies would have much greater value, but this is unfortunately beyond the scope of our current paper, and we, therefore, aim to address it in our future work.
2. I think that two subpopulations with different mutation rates can also occur without stress response, for examples see theoretical study by Lobinska et al Genetics 2023 and refs within. Does your method accommodate such cases? The reviewer is correct that mutation rate heterogeneity can arise even without stress responses. In principle, phenotypic switching, as described in Lobinska et al., can be accommodated by our heterogeneous model. Unfortunately, however, mutation-rate estimation using our method would not work well in the scenario described in the study by Lobinska et al. That is because they assume that the mutator phenotype is neutral, which corresponds to a relative fitness=1 in our model. As we show for this scenario, parameter estimation is biased (Results section 3.1, Figure 4) and heterogeneity in mutation rates cannot be detected through fluctuation assays (Results section 3.4, Figure 5). Therefore, we do not refer specifically to the Lobinska study in our manuscript.
 3. I think it would have been better to first describe the full model and then describe in a separate section the simple model used for inference – and make it explicit using equations which assumptions you make (e.g. do you set rho=1? In one point you make it explicit in line 222 but I would appreciate making things more explicit in other places, too) and where stochastic processes are involved. The way things are, it is confusing to me and I doubt I could reproduce either model just from the text. We followed the reviewer’s suggestion and restructured the Model & Methods section, first introducing the exact model (in section 2.3.1) and then describing the approximations we make to derive the mutant count distribution we use in the inference (in section 2.3.2).
4. It is especially unclear to me how the stochastic simulation works. You mention in some places a branching process but I don’t think I saw a clear and formal description on how it works – the equations seem to focus on a deterministic model and the approximations required for the max likelihood estimation. Do you use Gillespie algorithm? Moran process? SDE? Something else? We apologize that this information was previously missing in the manuscript. We now explicitly write out the stochastic events in Equations 2 and 12. We also extended the Simulation methods section 2.5.1, including the details about the stochastic simulation of mutant dynamics (we use Gillespie’s algorithm indeed).
5. I think the methods section should start with a clear definition of how the fluctuation assay works, as it is not explicitly defined. There’s a growth phase (which is standard fluctuation tests does not include selection but to infer SIM it does) followed by a selection phase in which mutants that occurred in the growth phase are selected using some stressor (the original Luria-Delbruck used phages right? And now days people usually used some rifampicin and other drugs?). I’m not sure I managed to understand all the details. Maybe even add an illustration of the experiment…? In the introduction, we extended the explanation of the experimental setup and protocol of fluctuation assays, beginning in Line 64. We decided not to include an illustration of the experiment because fluctuation assays are already widely used in the field, but we now refer to an exemplary protocol (https://www.jove.com/es/t/60406/measuring-microbial-mutation-rates-with-the-fluctuation-assay).
6. Model selection - it seems strange to me that you use AIC when you can use Cross-Validation: you use simulations so you have as much data as you want; and even when using real experimental data the experimenter is likely to have dozens of replicates. See Arlot & Celisse 2010 10.1214/09-SS054. I would at least like to see if another model selection approach performs better than AIC or if it is just not possible to select between the models (which may be the case, unfortunately). Please see our answer below, where we address both the reviewer’s comment 6 and 7.

7. Model selection 2- is the homo-response model not nested within the hetero-response? That is, if you set parameters of the hetero-response model to some fixed values, can you get to the homo-response model? Maybe f_on=0…? If so, you could use the likelihood-ratio test (Wilks test), which in many cases is stronger than information criteria like AIC. Motivated by the reviewer’s comments, we tested alternative model selection procedures and eventually chose a procedure that involves two steps.

(1) For the first model selection step, thanks to the reviewer’s suggestion, we now use a likelihood-ratio test to select the best model version within the set of homogeneous and within the set of heterogeneous models since each set is, separately, nested.

Unfortunately, however, none of the homogeneous-response models is nested within any of the heterogeneous-response models. This is because our heterogeneous model assumes that the per-division mutation rate of response-off cells is the same under permissive and stressful conditions, whereas the homogeneous model considers two different mutation rates under permissive and stressful conditions. Therefore, we cannot use LRT to compare homogeneous and heterogeneous-response models.

(2) Instead, for the second model selection step, to compare between homogeneous and heterogeneous-response models, we tested implementations of AIC, BIC and cross-validation (10-fold with modified one-standard error selection rule as described in https://esajournals.onlinelibrary.wiley.com/doi/full/10.1002/ecm.1557) and performed model selection for simulations over a range of parameters, simulating under
(i) the exact heterogeneous-response model for a range of relative fitness of response-on cells and different numbers of parallel cultures,
(ii) the homogeneous-response model with constrained mutant fitness cost for different increases in population-wide mutation rate and
(iii) the homogeneous-response model with mutant cost only under stressful conditions (i.e. unconstrained mutant fitness) again for different increases in population-wide mutation rate.

We present the results with model selection using either AIC (as before) or BIC (new) in SI section S14. Comparing AIC and BIC, neither outperforms the other in all scenarios. For example, the BIC has a higher rate of true positives than the AIC in case (i) (SI Figure S13), but at the same time, its rate of false positives in case (iii) is higher, too (SI Figure S15). To us, it seems more important to reduce the number of false positives, and therefore, we use the AIC for the results shown in the main manuscript. However, we acknowledge that a case could be made for either approach, and therefore also show the results using the BIC in the SI section S14 and provide our code in case another user prefers to use BIC in their application.

We also tested cross-validation, which we present alongside the AIC and BIC results in Figures 1-3 at the end of this document, but do not present in the manuscript. Overall, we find that cross-validation performs qualitatively similarly to BIC, yet worse in all cases except (iii) for small increases in mutation rate, where it performs equally well (Figure 3C1 and 3C2 below). Moreover, despite cross-validation being universally applicable, we think it is not particularly suitable for our specific model selection problem. Cross-validation involves splitting the data into a training set for model fitting and a small validation set to assess the ‘prediction loss’ of the considered model. In our case, this implies first estimating model parameters using the mutant count distributions with a small number of parallel cultures removed from the data set and then predicting the number of observed mutant colonies of the previously removed parallel cultures. However, due to the stochastic nature of the process, the prediction loss will generally be very large for all compared models, and therefore, model selection often inconclusive, in particular for small numbers of parallel cultures (c). This is confirmed by the worsening performance of CV when we test it on decreasing (c = 50, 20, 10) shown in Figure 1 below. Given that many experiments are carried out with smaller *c,* we preferred model selection methods that are likely to perform well on a wider set of existing fluctuation assay data.
8. Did you check what happens if you simulate from the homo-response model and infer both homo- and hetero-response models? In that case, what is the rate at which you will select the wrong (hetero) model? Following the reviewer’s suggestion, we simulated under the homogeneous model and performed model selection, using our new model selection procedure. We present these results in SI sections S11 and S14. In summary, we find that when simulating under a homogeneous model with mutant cost constrained to be equal under permissive and stressful conditions, the number of false positives is very small. For simulations without the constraint on the mutant cost, however, the number of cases where no model or the wrong (heterogeneous) model is selected increases with the severity of the mutant cost and with the magnitude of the increase in mutation rate that is simulated.
9. When the method fails to choose the correct (hetero) model, is it because you infer f_on? if you use the correct f_on rather than inferring it, thus having less parameters (k), will you make the correct model choice? We agree in principle: Using the correct f_on reduces the number of inference parameters of the heterogeneous model and would thus decrease its AIC, which would likely increase the number of cases in which this model is selected. However, we decided not to do this analysis because we think that model selection (between homogeneous and heterogeneous responses) is not required anymore when stress responses are already known to be heterogeneous, which is necessarily the case if an estimate of f_on is available. Therefore, we keep our Results (section 3.4) focused on the case where the presence of heterogeneity (and hence f_on) is unknown and ask whether we can infer heterogeneity through model selection.
10. You perform the estimation on two datasets – from permissive and stress growth phases. How about three or more datasets? You can do one experiment without stress in growth phase, one with rifampicin, one with nalidixic acid, one with high salt, etc etc. These experiments may differ in rho and mu (and maybe other parameters) but might help to better estimate and select. Just an idea… As the reviewer correctly realised, different stressful conditions would typically yield different values in most of the model parameters. Therefore, we do not expect to be able to improve our estimation of these parameters (e.g. rate of switching on the stress response or associated mutation-rate increase) by inferring jointly from data under multiple different stressors tested in parallel. The only parameter that we expect generally to remain the same would be the mutation rate of response-off cells (mu_off, assumed equal to the mutation rate in permissive conditions). In the case that several multiple stressors had been tested in parallel in an experiment, we would then recommend jointly estimating mu_off on all datasets to ensure that the inferred value is consistent. Although the reviewer raises a valid point, we believe it only becomes relevant when considering experimental data where multiple different stressors have been considered, which is beyond the scope of our study. On the balance, we felt this would require a lot of additional explanation and would risk adding confusion by raising this point in the Discussion, since we do not present any results for more than one stressor. Therefore, we decided not to address this point in the manuscript, but we thank the reviewer for suggesting it and will keep it in mind for future work.
11. It is not clear to me why it is interesting to estimate delta-mu-hat and compare between the two models. Can you explain in the text? Maybe I missed it. We agree with the reviewer that not much information can be gained from this comparison and have removed it.
12. I think you can call H the mutation supply ratio. Because the mutation supply of each subpopulation is N x f x mu where N is population size, f is the frequency of that subpopulation, and mu is the mutation rate of cells in that subpopulation. Mutation supply is a commonly used concept, see 10.1126/science.283.5400.404, 10.1093/g3journal/jkac190, 10.1111/ele.13922. It also makes a lot of sense – when mutation supply of on-cells is high enough, their effect can be detected, otherwise they cannot. We thank the reviewer for this suggestion and are now using the term mutation supply ratio, denoted S (introduced in Equation 27, section 2.3.2).
13. Something missing in this paper is an estimation of the uncertainty of the maximum likelihood estimates; I do not mean what’s shown in Fig 2C (which is fine) but rather confidence intervals or a similar quantity, especially around mu_on/mu_off. If such a quantity was estimated, it could have also been used instead of model selection: if the value 1 is outside the confidence interval then we can say that there is significant heterogeneity. Similarly for f_on and 0 if f_on is estimated. We agree that an estimation of uncertainty of the maximum likelihood estimates was missing and have implemented 95% confidence intervals using a profile likelihood approach, described in Methods section 2.4.2. In the SI (Figure S4), we show that for the purpose of evaluating the accuracy and precision of our new method, the median width of confidence intervals gives the same qualitative picture as our approach shown in Figure 2. Specifically, the coefficient of variation of the point estimates across the R=100 simulations (Fig. 2C) shows a similar qualitative relationship with parameters - the relative switching rate and the mutation-rate increase - as the median width of the respective confidence intervals (Fig. S4C). Confidence intervals can also be used as additional information in the model selection but cannot replace model selection entirely. That is because the homogeneous response model with mutant fitness cost and the heterogeneous model can produce similar patterns in fluctuation assay data. In such cases, both models would fit the mutant count data equally well, representing alternative explanations that cannot be distinguished. Therefore, we have not incorporated a comparison of CIs into our model selection.

**Minor comments:**
14. Line 56: on SIM facilitating complex adaptation and its evolution due to its effect on adaptive evolution see Ram & Hadany 2012, 2014. We thank the reviewer for pointing us to these theoretical studies and added the references in the Introduction (Line 58).
15. Line 99: I wonder “within population heterogeneity” is better than “cell-to-cell heterogeneity”? We agree that “within-population” fits better here (Line 105) and changed the phrasing in most cases throughout the manuscript.
16. Line 144: in *some* of the cells, right? Unfortunately, we are unsure which sentence the reviewer is referring to, as Line 144 in the original manuscript does not refer to cells.
17. Line 139: alpha is defined in line 131, consider reminding us what it is after eq 2 We now remind the reader of the meaning of alpha at this point (Line 232).
18. Line 165: this is confusing, what do you mean by “end of the growth phase”? In a fluctuation assay, the parallel cultures are first grown for a certain period, typically overnight, before they are plated onto selective media. We call this first part the “growth phase”, and the time after growing and before plating is the “end of the growth phase”. For clarity, in the Introduction, we now define the term “growth phase” when we first describe the fluctuation assay (Line 67).
19. Line 177: Is t_f the time duration used for approximating f_on* using f_on(t_f) ? Maybe this confusion goes back to line 112 – you could be more explicit about explaining how a fluctuation assay is designed (even if you in line 63…) The reviewer is correct, we use f_on(t_f) as an approximation for f_on*. We now explicitly explain this in section 2.3.2, Lined 283f. As suggested, we also extended the description of the experimental setup and protocol of fluctuation assays in the Introduction (Lines 64ff).
20. Line 181: “on the plates”—this again goes back to defining the fluctuation assay more clearly We have clarified this in our extended description of fluctuation assays in the Introduction (Lines 64ff).
21. Line 186: it is unclear how you implement an “altered division rate” What we mean by “altered division rate” is simply that the division rate has a different value. We have removed the unclear wording “altered division rate”.
22. Line 188: what is rho? It is not defined…. Until line 209. Then, does “differential fitness” mean “relative fitness” or something else? The reviewer is correct that “differential fitness” means relative fitness, i.e. (division rate – death rate of mutants)/(net population growth rate of non-mutants). We have restructured the Model & Methods such that the symbol rho does not appear before it is properly defined (in Line 167) anymore.
23. Line 190: you say “the two-type branching process described above” but you did not formally describe a branching process. You formally described an ODE system and then verbally mentioned a branching process. It would be good to formally describe what you mean when you say “branching process” – we don’t need an introduction to branching processes (Harris 1969) but we do need the formal equations so that we know exactly what you mean. As the reviewer suggested, we added a formal description of the branching processes used in the models, including formal equations, in section 2.1 (Equation 2) and section 2.3.1 (Equation 12).
24. Line 195: missing “see below”? We have restructured the Model & Methods section such that the sentence referred to here now appears within section 2.3.2 (Line 310), where we provide the full explanation and no longer need to say ‘see below’.
25. Line 210 : consider if you think it would be good to mention that z is a latent variable and does not have a meaning (its not the number of mutations etc) for the sake of those that don’t remember what a pgf is. It can be confusing. As the reviewer suggested, we added a sentence where we first introduce the PGF (section 2.1, Equation 3) explaining that G(z) does not directly give the probability of observing a specific mutant count, and that z is a dummy variable (Line 172).
26. Line 211: is the recursive expression in p_m? that is, p_m = f(p_m-1)? In G(z)? The reviewer is correct that the recursive expression is in p_m=f(p_m-1). However, we have removed the details about how p_m is calculated from the manuscript to avoid confusion, as it is only relevant so that we can calculate log-likelihoods in a numerically feasible manner. We cite the reference where details can be found (<https://iopscience.iop.org/article/10.1088/1742-5468/2015/01/P01011/meta> in Line 174).
27. Line 218: why is this important? The assumption that all mutant cells form a visible colony is important because visible colonies on selective plates are how mutants are counted, and failure to form a colony would thus lead to underestimation of the mutation rate. This assumption is made in most estimation methods. In the restructured version of Model & Methods, this sentence appears now in the description of the standard model of fluctuation assays (section 2.1, Line 149).
28. Line 223: it results in a probability-generating function, not in a mutant count distribution; I know the former represents the latter, but I think that for most readers, when they are told the following expression is a distribution, they expect to see pmf or cdf, not a pgf. Maybe you want to remind the readers that p_m = G^(m)(0)/m! (the m-th derivative of G at zero divided by factorial m, right?) We have clarified (now Line 217) that we are showing PGFs, as the reviewer correctly commented, but we decided not to include the definition of the PGF because the use of the PGF is not a novel part of our work and we do not believe it is essential for the reader’s understanding of our methods. We have cited https://iopscience.iop.org/article/10.1088/1742-5468/2015/01/P01011/meta for a detailed description of how the PGFs are used (Line 174).
29. Line 238, 262 missing “-“ in pgf? Actually, this is correct as is; the minus sign dropped out with the logarithm.
30. Eq 20-21 missing “(z)” We thank the reviewer for pointing out the missing (z) which we added (now Equations 5 and 6).
31. Line 251: does that mean you use data from two experiments, one with growth phase without a stressor and one with growth phase with a stressor, and infer the parameters once using both data? I think it can be phrased a bit clearer… We indeed use data from two experiments which differ in the condition of the growth phase (one without and one with a stressor). This concept is crucial for understanding our new method, and we now explain it in the first sentence of the Model & Methods section (Line 117).
32. Eq 22: likelihood functions are usually defined as L(theta | X) = p(X|theta); are you sure about how you define it here? Seems reverse. Also, this is the likelihood or the log-likelihood? Because line 258 says it is log-lik but I don’t see why you need to say that here (except that eq 26 uses log-lik as L). Also line 259 says “that is a measure of the probability…” but that’s not really correct – it is not a “measure”, the lok-likelihood is defined as the log-probability of observing… or am I wrong? The log-lik is also not the probability but the log-probability… We thank the reviewer for correctly pointing this out. We have corrected the respective statements in the manuscript (section 2.4, Lines 337ff).
33. Eq 22: more importantly, you did not show us the likelihood function here, but only say use the “count distributions” defined above (although you explicitly define the count distributions’ generating functions, and not the probabilities themselves. That’s ok, but I think it might be clearer to lay out how L is defined in terms of p_m, and then explain p_m is defined using G(z) and the recursion from [30]. As the reviewer suggested, for clarity, we now explicitly define the likelihood function in terms of p_m (section 2.4, Equation 29).
34. Line 261: what are the parameters inferred? You group them in theta, but can you be explicit and mention which parameters exactly are inferred? The inferred parameters depend on the specific model used in the inference, which is why we do not explicitly mention them here but group them in theta. In the following section (2.4.1 Parameters in the inference), we describe which parameters are inferred in each model. To have all the information in one place, we have added Table 1 with a summary of the inference parameters.
35. Line 263: which optimization algorithm do you use? What kind of fine tuning or hyper parameters have you used? We use the default option implemented in the Julia package Optim.jl https://julianlsolvers.github.io/Optim.jl/stable/ and have specified this now in the manuscript (Line 354). For any details on fine-tuning and hyper parameters we refer the reviewer to the repository <https://github.com/LucyL-J/Quantifying-SIM> with a complete documentation of all inference algorithms (in Line 360).
36. Line 265 “the initial parameters for the optimization *algorithm*”; what do you mean “among others”? For some parameters, we calculate the initial values using the probability generating function method described in <https://www.ncbi.nlm.nih.gov/pmc/articles/PMC4632052/>; for others, we set the initial value to our null expectation, for example, to one for the differential mutant fitness. To avoid confusion, we removed the sentence from the manuscript and refer to the repository for details (in Line 360).
37. Line 275 – do you mean that on-cells might mutate without dividing? Thank you for raising this important point. That is correct, but we realised that we did not clearly explain how this could be the case. Reviewer 1 raised a similar point; please refer to our response to their comment 4 earlier for our full explanation. To summarise, chromosomes continue to be replicated (with the possibility of mutation) even though the cell is not dividing, and we now cite experimental evidence (Bos et al. 2015) to support our model of how mutants arise. We have added this explanation to the manuscript in section 2.3.1 under “Mutant population dynamics”, Line 271ff.
38. Line 281: do you mean that r_on=1 ? how can it be zero? Does zero mean that you assume that growth rate of on-cells is zero? The default value is indeed r_on=0, and the reviewer is correct that this implies a zero growth rate of response-on cells. Without cell death, this means that cells are not dividing (a reasonable first approximation for the SOS response; see our Introduction), but can still generate mutations, as we now clarify (see our reply to comment 37 above).
39. How can f_on be measured experimentally? Can you explain or give a reference? This can be done by engineering a fluorescent reporter for the stress response and performing either microscopy or flow cytometry. We added references for both approaches in this sentence in the Discussion (Line 787f).
40. Line 296 – what’s the difference between the stationary fraction and the fraction at the end of the growth phase? Is the latter not the former? The fraction at the end of the growth phase is a good approximation of the stationary (i.e. equilibrium) fraction. However, since in the simulations we set the initial fraction of response-on cells to f_on(0)=0, it takes some time for the response-on subpopulation to approach the stationary fraction, especially when the stationary fraction is comparably large (which is the case when the relative fitness of response-on cells is not small). In section 2.3.2 ‘Derivation of the mutant count distribution’, when we introduce the stationary fraction (Equation 15), we now explain the relation between stationary fraction and fraction at the end of the growth phase (Lines 283f).
41. I think it would be good to highlight which parameters, if any, are shared between the hetero- and homo-response models – as you estimate both; and between the permissive and the stress environment, as you perform the inference jointly on results of the two experiments. We agree with the reviewer’s point and have added Table 1, which summarises the inference parameters, including under which model(s) they are used, to which condition they apply (permissive/stressful), and if they are inferred separately or jointly.
42. Line 319 – maybe start by saying you choose parameter values, then simulate synthetic data, then infer and compare to true parameter values (which you know because you chose them to begin with) We rephrased the first paragraph of this section (now 2.5) in the way suggested by the reviewer.
43. Line 324 – how do you integrate the likelihood over the c=50 parallel cultures? Why do you use 50? Because of 96-well plates? Since the parallel cultures are independent of each other, we can multiply the respective (log-)probabilities to obtain the log-likelihood. In section 2.4 ‘Model fitting and parameter estimation using maximum likelihood’, we now explicitly define the log-likelihood as a function of the log-probabilites to clarify this approach (Equation 29). And yes, c=50 is chosen such that the experiment can easily be fitted in a 96-well plate, along with additional spare cultures to estimate the final population size. We now explain this decision and cite an experimental protocol (https://pubmed.ncbi.nlm.nih.gov/31840662/) in section 2.5.1, Lines 467f.
44. Line 333: I don’t think the word “underlying” is needed here; instead, as I mention above, I would suggest you explicitly mention you choose parameters and therefore you know what the true values are and so you can compare estimations to true values. We agree with the reviewer and removed the term “underlying” in this sentence (now 473).
45. Eq 24 – why compute the error of the median estimate across R replicates to the true value rather than the median of R errors? The latter seems more useful to me, as in reality when I use your method, I will have R=1… It’s your call but the median of the error seems more useful to me. You can then also compute 95% intervals, not just the median. We agree with the reviewer that the median of R errors is more useful and now plot this quantity instead. This would indeed allow us to compute 95% intervals, but we decided to stick with our boxplots (e.g. Figure 2A) as they present the same qualitative result.
46. Did you explore the effect of c on estimation accuracy and model selection? Inspired by the reviewer’s comment, we have explored the effect of c on estimation accuracy and model selection and present the results in the SI (Figure S3). In short, for smaller c, estimation remains accurate (i.e. unbiased), but some precision is lost, and the number of cases in which no model is selected increases.

47. Fig 2: why is the x label in panel A different from panels B and C? We changed the label in Figure 2B to be consistent.
48. Fig 2: it seems RE and CV are correlated, is that right? We think the reviewer is correct, but we have not statistically tested this hypothesis as we believe the visual results in Fig. 2 are sufficient to make our points about accuracy and precision.
49. Line 439: this effect is seen in Fig 2B and C right? There’s a quick improvement of the estimation when mutation rate increase x relative switching rate > 0.5 or something like that… So, I wonder if you can connect H and Fig 2 – either rephrase H in terms of relative switching rate or change Fig 2 to have H or f_on in it…? The reviewer’s intuition is correct, and we followed the suggestion to rephrase H (now called the mutation-supply ratio, S; see Equation 27) in terms of the relative switching rate. This now connects S to Figure 2, as explained in the text (Results section 3.1, Line 575).
50. You say that if the mutation rate increase is estimated to be >100 it is likely >25; can you say something opposite? If its estimated to be < 20 can then it is likely ~1? Or something like that?
Yes, along the lines suggested, we added the following statement to the paragraph (now Lines 570f): “If the mutation-rate increase is estimated close to zero (<10^-2), it is very likely to be <10.
51. It’s a bit strange that in Fig 2 the estimate can be lower than 1; is that correct? Can you not assume it is >=1? Would that matter at all for the inference? Can we say that if we estimate <1 than we know the estimation is wrong? In fact, H<1 implies that the contribution to the mutation supply from the response-on subpopulation is comparably small (smaller than from the response-off subpopulation), but not that it is absent. Therefore, we cannot conclude that the estimation is wrong when H<1. We added a sentence after introducing H, emphasising that H=0 (now called mutation-supply ratio and denoted S) implies the absence of heterogeneity (Line 326). In our inference, we do constrain H to be >=0.
52. I wonder if you can use statistical calibration to correct the estimation errors when H<<1, see <https://doi.org/10.2307/1403690>. Just an idea… We thank the reviewer for the suggestion, but we think a calibration approach is not suitable in our case because we have only one ‘measurement’ of the mutation rate per assay (i.e. in either permissive/stressful condition), not one per parallel culture.
53. Line 473: do you mean they do not divide in the growth phase or the selection phase or both…? We thank the reviewer for pointing out the ambiguity. What we mean here is during the growth phase and we added this in the sentence (now line 621).
54. Line 525: you compare model fit or AIC? We compare AIC and have corrected this sentence (now Line 680).
55. Fig 5: how many simulations did you run (what is R)? Throughout the manuscript, for each parameter combination, we run R=100 simulations. We mention this in section 2.5.2 of Model & Methods (Line 474), where we describe the simulations, and again at the beginning of the Results section (Line 537). We decided not to mention it again in each of the figures to avoid repeating ourselves.
56. Line 532: I suspect that the effect of alpha and f_on would be similar – low alpha and f_on would lead to selecting the homo-response model; high alpha / f_on would lead to selecting the hetero-response model; is that right? We thank the reviewer for this interesting question. In fact, the effect of alpha and f_on on the model selection is somewhat different: For small alpha (resulting in small f_on), there is effectively no detectable increase in mutation rate. In this case, no model is selected because both homogeneous and heterogeneous models estimate no increase in mutation rate (corresponding to M=1 and S=0, respectively) and, therefore, fit the data equally well. For intermediate alpha, the heterogeneous model is selected in around 75% of the simulations (as shown in our Results Figure 5A for r_on=0). Unfortunately, for a larger alpha (and with it larger f_on), the model selection does not improve further. This is because, independent of alpha, the homogeneous model with mutant fitness cost and the heterogeneous model represent alternative explanations for the observed mutant count data. We added a Figure to the SI (Figure S11).
57. Do you use c=50 in the model selection section? Would model selection work better if you use c=500? c=5000? The reviewer is correct that, in principle, model selection works better for larger c because the observed mutant count distribution will be a better representation of the true distribution. However, there are two limitations: (i) We made some approximations to derive an expression of the mutant count distribution (described in section 2.3.2), implying that even for c -> Infinity, we could not capture the exact distribution. (ii) For larger c, the probability of observing a “super-jackpot event” with thousands of mutants becomes large, which gives rise to numerical instabilities in calculating the probability distribution. More importantly, however, our question in this section (Results 3.4) was to determine whether it is possible to select between models in a realistic experiment, with an experimentally feasible number of parallel cultures. Therefore, we restricted our analysis to values of c<=50.
58. Line 565: there has been evidence that only a subpopulation induced a stress response for a while now, since at least González et al 2008, see for example 10.1016/j.molcel.2019.02.037 and 10.1371/journal.pgen.1000208. We thank the reviewer for pointing us towards more evidence of stress-response induction in only a subpopulation and added Pribis et al. 2019 as a reference (Introduction, Line 90). We decided not to cite Gonzalez et al. 2008 because we restrict ourselves to reference single-cell studies in this context.
59. Line 570: if someone jumps from introduction to discussion (likely for a microbiologist without mathematical background), they will not know what mu and f_on are. We acknowledge the reviewer’s point and now verbally introduce mu and f_on again at the beginning of the Discussion (Lines 742, 745).
60. Line 575: another option is to test different stressors, right? due to differences in either alpha -> f_on or in mu. But the problem is that if you want to know about mutation rate heterogeneity in a specific environment – e.g. for clinical reasons – then increasing the drug concentration or changing the drug doesn’t help you. What else can you do? Increase c? Increase t_f? Thank you for this thought-provoking question. We expect that the mutation-rate increase associated with the induction of the stress response will be (approximately) independent of the stressor used to induce it, i.e. the specific drug or its concentration. On the other hand, we expect the rate of induction (the switching rate) depends on the drug concentration. Therefore, the mutation-rate increase associated with a particular stress response could be measured from fluctuation assays conducted at higher drug concentrations to improve the quality of the estimation: as we show in our Results (Figure 2), accuracy and precision of the estimate improve with higher switching rate, alpha. The estimated mutation rate could then still be applied in a model with a different drug concentration or even a different stressor, provided it induced the same stress response. We explain this briefly in the Discussion (Line 750ff). Increasing the number of parallel cultures also improves the estimation; this can be seen when comparing our new results with smaller numbers of parallel cultures to our previous results with c=50 (SI Figure S13).

Unfortunately, we do not see how changing the duration of the growth phase (t_f) could improve the estimation because if a too-small S is the reason for poor estimation, increasing t_f would not increase S, which is independent of t_f.

61. Line 587: why “formal”? what’s “informal model selection”? I think you can remove this word. We agree with the reviewer and have removed the term “formal” (now Line 760).
62. Line 590, 602: “preferred”->”selected” (otherwise not clear preferred by whom…) We rephrased this sentence as the reviewer suggested, and now stick to the term “selected” model throughout the manuscript (now Lines 764 and 782).
63. Line 598: this suggestion is kind of weak, as the parameter rho is an “effective parameter” and it would be hard to determine if fitness values of individual clones are “reasonable” compared with the estimated rho value. We apologise for not being clear about which kind of fitness measurement we were referring to. We suggest that the relative fitness of mutants compared to non-mutants were measured in competitive fitness assays, once under permissive and once under stressful conditions, Then, it would indeed be possible to assess whether estimated rho values, in particular the difference between permissive and stressful, are reasonable. In the revised manuscript, we clarified which fitness measurement we had in mind in line 776.
64. Line 594: I think “neglected” is a better word than “overlooked.” We think that, in this context, “overlooked” fits better than “neglected” because we aim to point out the unawareness towards heterogeneity rather than active decisions to neglect it. Therefore, we decided not to change our wording in this instance.

65. Line 607: I don’t understand the suggested approach – engineer reporters where? In the genome? Where in the genome? Please explain and add a reference; do you mean something like Woo et al 2018 (10.1126/sciadv.aat1608)? We are indeed referring to an approach used by Woo et al. 2018, among others. We added a couple of references in this sentence (now Lines 785ff).
66. Line 627, 631: see also Ram and Hadany 2012, 2014 for models of SIM. We thank the reviewer for pointing out these theoretical studies. We have now cited them in the Introduction where we state: “It has been proposed that SIM in bacterial cells could accelerate the evolution of populations that are poorly adapted to their environment” (line 58). However, we do not cite them again in the Discussion at the point the reviewer mentioned, because they do not specifically support the statement we made there. We have now slightly edited the relevant sentence (now line 808, change indicated here in bold) to clarify the distinction: “Theoretical modelling suggests that single-locus adaptation can be accurately captured by the population mean mutation rate, but within-population variation **(even for a fixed population mean)** can speed up multi-locus adaptation Alexander el al. 2016.” Ram & Hadany did not draw their comparisons at a fixed population mean mutation rate, but rather, for absence versus presence of stress-induced hypermutation.
67. Line 655: do you think it would be possible to distinguish between hetero and homo-response if using single-cell or pooled sequencing rather than just plating on selective media? We have thought a lot about this interesting suggestion. Pooled sequencing of the mutant colonies on a selective plate could identify the relevant mutations and give an indication of whether the mutants descended from the same or multiple independent mutation events, and the extent of genetic diversity might, in principle, give a signal of underlying population heterogeneity. We believe a formal analysis (which is outside the scope of our study) would be needed to give a definitive answer. However, unfortunately we expect that sequence analysis would bring the same model selection challenges as the usual inference based on mutant counts. We can find parameters where both the population mean mutation rate (hence the expected number of mutation events per culture) and the mutant count distribution are very similar between homogeneous and heterogeneous response models; see our Figure S2 for an example. We expect that both models would lead to similar patterns of genetic diversity and could, therefore, not be distinguished. For that reason, we have decided not to mention sequencing as an additional/alternative approach for model selection.

Figure 1: **Model selection when simulating under the heterogeneous-response model.** We use the same simulation parameters as in Results Figure 5 and use a two-step model selection procedure with LRT as the first step and either AIC (**A**), BIC (**B**) or cross-validation (**C**) as the second selection step, and c=50 (**1**), c=20 (**2**) or c=10 (**3**) parallel cultures in the inference.

Figure 2: **Model selection when simulating under the homogeneous-response model with constrained mutant fitness.** We use a two-step model selection procedure with LRT as the first step and either AIC (**A**), BIC (**B**) or cross-validation (**C**) as the second selection step, and simulate with an increase in population-wide mutation rate of mu^s/mu^p=3.2 (**1**), mu^s/mu^p =10 (**2**) or mu^s/mu^p =32 (**3**). The parameter range used in the simulation is rho^p=rho^s in [0, 1].

Figure 3: **Model selection when simulating under the homogeneous-response model with mutant fitness only under stressful conditions.** We use a two-step model selection procedure with LRT as the first step and either AIC (**A**), BIC (**B**) or cross-validation (**C**) as the second selection step, and simulate with an increase in population-wide mutation rate of mu^s/mu^p=3.2 (**1**), mu^s/mu^p =10 (**2**) or mu^s/mu^p =32 (**3**). The parameter range used in the simulation is rho^p=1, rho^s in [0, 1].
